# Trabecular Bone Structure Correlates with Hand Posture and Use in Hominoids

**DOI:** 10.1371/journal.pone.0078781

**Published:** 2013-11-14

**Authors:** Zewdi J. Tsegai, Tracy L. Kivell, Thomas Gross, N. Huynh Nguyen, Dieter H. Pahr, Jeroen B. Smaers, Matthew M. Skinner

**Affiliations:** 1 Department of Anthropology, University College London, London, United Kingdom; 2 School of Anthropology and Conservation, The University of Kent, Canterbury, United Kingdom; 3 Department of Human Evolution, Max Planck Institute for Evolutionary Anthropology, Leipzig, Germany; 4 Institute of Lightweight Design and Structural Biomechanics, Vienna University of Technology, Vienna, Austria; 5 Department of Anthropology, Stony Brook University, Stony Brook, New York, United States of America; Museo Nazionale Preistorico Etnografico ‘L. Pigorini’, Italy

## Abstract

Bone is capable of adapting during life in response to stress. Therefore, variation in locomotor and manipulative behaviours across extant hominoids may be reflected in differences in trabecular bone structure. The hand is a promising region for trabecular analysis, as it is the direct contact between the individual and the environment and joint positions at peak loading vary amongst extant hominoids. Building upon traditional volume of interest-based analyses, we apply a whole-epiphysis analytical approach using high-resolution microtomographic scans of the hominoid third metacarpal to investigate whether trabecular structure reflects differences in hand posture and loading in knuckle-walking (*Gorilla*, *Pan*), suspensory (*Pongo*, *Hylobates* and *Symphalangus*) and manipulative (*Homo*) taxa. Additionally, a comparative phylogenetic method was used to analyse rates of evolutionary changes in trabecular parameters. Results demonstrate that trabecular bone volume distribution and regions of greatest stiffness (i.e., Young's modulus) correspond with predicted loading of the hand in each behavioural category. In suspensory and manipulative taxa, regions of high bone volume and greatest stiffness are concentrated on the palmar or distopalmar regions of the metacarpal head, whereas knuckle-walking taxa show greater bone volume and stiffness throughout the head, and particularly in the dorsal region; patterns that correspond with the highest predicted joint reaction forces. Trabecular structure in knuckle-walking taxa is characterised by high bone volume fraction and a high degree of anisotropy in contrast to the suspensory brachiators. Humans, in which the hand is used primarily for manipulation, have a low bone volume fraction and a variable degree of anisotropy. Finally, when trabecular parameters are mapped onto a molecular-based phylogeny, we show that the rates of change in trabecular structure vary across the hominoid clade. Our results support a link between inferred behaviour and trabecular structure in extant hominoids that can be informative for reconstructing behaviour in fossil primates.

## Introduction

Understanding the functional significance of skeletal morphology plays a critical role in addressing fundamental questions of primate evolution and particularly questions of human evolution. Traditionally, functional interpretations of skeletal or fossil remains have been based on external morphology and, although informative, researchers argue over which features are functionally relevant for reconstructing behaviour in the past *versus* features that are possibly primitive retentions and no longer ‘functionally important’ (see review in [1]). Such debates have profound effects on our reconstruction of behaviour in fossil ancestors and the evolutionary pathways of humans and other primates. Cortical and trabecular bone remodel throughout life in response to mechanical stress [2]–[5] and, as such, can provide more direct insight into the function of a particular bone, joint and/or morphology than can be gleaned from external morphology alone. In short, analyses of internal bone structure can offer insight into what an individual was *actually* doing versus what they may have been *capable* of doing [6], potentially providing resolution to many longstanding debates in human and primate evolution.

The general concept that bone adapts to mechanical stress during life is broadly known as “Wolff's law” [7] or “bone functional adaptation” [4] and is a fundamental assumption of all palaeoanthropologists trying to reconstruct behaviour in the past [4]. Although there is a genetic influence to the underlying structure [2] and debate regarding how well bone adapts at different stages of ontogeny [8], much experimental and comparative evidence supports the concept that both cortical and trabecular bone can respond to local stress and adapt to their mechanical environment [3]–[5]. Bone can be removed (overall structure becomes weaker) where stress is lower and bone added (overall structure becomes stronger) where stress is higher to optimize the trabecular structure. Since trabecular bone remodels rapidly throughout life [9], its structure can offer a more direct window into an individual's behaviour and, in particular, to joint posture during predominant stress [3], [5].

Several studies have looked to trabecular bone to identify behavioural signals – either locomotory or manipulatory – in humans and other primates that could then be applied to fossil specimens [10]–[25]. The majority of these studies have focused on the humeral and/or femoral head and generally have not found clear locomotor-related differences across non-human primate taxa [11]–[15], [21], [24], [25]. The poor correlation between trabecular structure and behaviour in these studies may be partly due to two factors: (1) the use of the traditional volume of interest-based approach, in which only a small subsample of trabecular structure in a given anatomical region is analysed and (2) the focus on anatomical regions of the limb that are further removed (i.e., more proximal) from the substrate (and thus from substrate reaction forces) than more distal regions of the limb (e.g. the hand or foot). To address these issues, we apply a new method [26], which enables analysis of trabecular structure throughout an entire epiphysis, to the third metacarpal head of extant humans and other apes. We investigate how variation in trabecular structure correlates with inferred variation in hand posture (i.e., different loading regimes) during locomotor behaviour (non-human apes) and manipulation (humans). If a strong correlation is found, such a result can provide more informed reconstruction of locomotor and manipulative behaviour in fossil hominins and other primate ancestors.

Extant apes exhibit a variety of locomotor and manipulative behaviours linked to their respective ecological niches, subsistence strategies and/or social organization, which require each to use their hands in different ways. Asian apes (*Pongo*, *Hylobates*, and *Symphalangus*), are all highly suspensory. *Pongo* engages in slow-moving, torso-orthograde locomotion, often supported by multiple limbs [27], [28] while *Hylobates* and *Symphalangus* are brachiators; a locomotor mode involving bimanual progression with a period of free flight [29], [30], [31]. During suspension and brachiation, the hand grasps the substrate in flexed-finger posture, with body mass (and the effects of gravity) below the hand [32], [33]. In contrast, the African apes (*Pan* and *Gorilla*), although they also engage in arboreal suspension and climbing, spend the majority of their locomotor time knuckle-walking, in which the dorsal surface of the middle phalanx contacts the substrate, with body mass (and the effects of gravity) above the hand [34], [35], [36]. Finally, the human hand is unique in being used primarily for manipulation and carrying involving flexed-finger hand postures, such as power and precision grips [37]. Compared to other apes, human hands are generally likely to incur a much lower magnitude of loading since the hands are not regularly used for locomotion and support of body mass [23].

Traditional methods of analyzing trabecular structure have been limited to quantifying the structure in only selected regions – a volume of interest – of a given bone or anatomical region. Although traditional methods have yielded important insights into behaviour in the past, functional signals are often obscure [11]–[16], [21], [25]. An alternative method, using a discriminant function analysis on a “suite” of trabecular parameters was able to distinguish some locomotor groups, but the functional significance of these variations in local trabecular structure remains unclear [24]. Such ambiguous results may partly stem from the fact that volume of interest-based methods overlook a substantial amount of information about function that can be gleaned from analyzing trabecular structure throughout the entire epiphysis. Furthermore, traditional methods suffer important challenges associated with quantifying regions of the bone that are anatomically and biomechanically homologous across different taxa, especially taxa that vary greatly in size or morphology [10], [38]–[40]. Trabecular parameters also can be sensitive to the size [38], [39] and location [38] of the volume of interest. Although previous researchers have taken various precautions to try to meet these challenges [11], [21], [24], [25], some methodological biases of using a volume of interest method cannot be removed (e.g., [41] discuss this issue of location and homology). Therefore, to address these issues, we apply a new method that quantifies trabecular structure throughout the entire epiphysis and enables visualization of variation in trabecular bone structure throughout the epiphyseal region. This whole-epiphysis approach has the potential to reveal patterns of trabecular bone structure and, in turn, behavioural signals, that cannot be observed using traditional volume of interest-based methods.

The link between trabecular structure and behaviour in the hands of extant apes is relatively unexplored. We suggest that certain joints of the hand may experience less complex loading (e.g., fewer tendon attachments, loading in fewer directions and/or a more limited range of motion) and incur substrate reaction forces more directly (i.e., more distal anatomical regions) relative to other joints in the skeleton (e.g. shoulder or knee). As such, these joints may exhibit variation in trabecular structure that can be more easily functionally interpreted. For example, the manual rays (i.e., metacarpals and phalanges) and specifically the third metacarpophalangeal joint, is one such region: only two extrinsic digital flexors (*Mm. flexor digitorum superficialis* and *profundus*) and *M. extensor digitorum* act on the joint and movement primarily occurs in one plane (flexion-extension). Previous studies have shown that there is potential for behaviour-related differences in the trabecular structure of the metacarpal using a single volume of interest-based approach [17], [22], [42], and backscattered electron imaging [23]. Of these, only two studies have investigated interspecific differences in the internal structure of the metacarpal head. Zeininger et al. [23] found differences in trabecular bone mineral density between *Homo* and a small sample of *Pan troglodytes* and *Pongo* that were consistent with increased bone remodelling in areas of predicted peak loading. A preliminary study by Zylstra [42] identified differences in trabecular bone volume fraction and degree of anisotropy between *Hylobates*, *Papio*, *Pan* and *Homo*, although details about the methods, sample size and results were not reported. We build upon this previous work using a larger and more diverse sample of hominoids and a more comprehensive analysis of the trabecular structure throughout the epiphyseal head. If a strong correlation can be demonstrated between different behavioural patterns and variation in trabecular bone morphology throughout the joint epiphysis, then this could be used to reconstruct locomotory and manipulatory behaviour in fossil hominoids and hominins.

In this study, we employ high-resolution microtomography (microCT) to determine whether variation in hand postures related to locomotor and manipulative behaviours in extant apes is reflected in the trabecular bone structure of the third metacarpal head. Since our sample is composed of extant hominoids, all of which are closely related, we apply a phylogenetically-integrated, variable rates approach to reveal evolutionary patterning of trabecular structure throughout the hominoid clade [43].

### Predicted Position of the Metacarpophalangeal Joint During Locomotion and Manipulation

The goal of this study is to determine if interspecific variation in trabecular bone structure of the third metacarpal head correlates with the habitual joint posture and loading of the metacarpophalangeal joint. Trabecular thickness quantified in this study is predicted to scale with body size, following Doube et al. [44] and Ryan and Shaw [45]. We test three main hypotheses, for bone volume fraction, trabecular bone distribution, stiffness (i.e., maximum Young's modulus) and degree of anisotropy following the findings of Zeininger et al. [23] and Zylstra [42], based on three basic categories of hand use and hand postures:

#### H1, Suspensory

**Figure 1:**
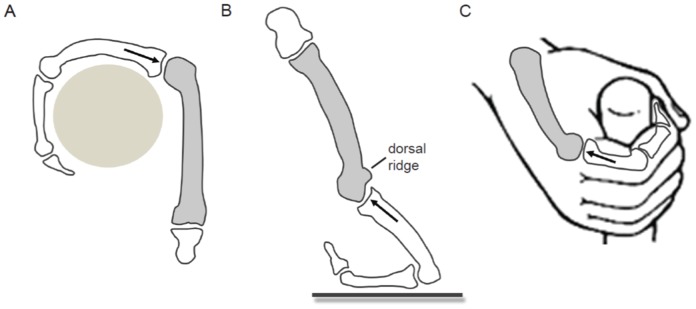
Predicted position of peak loading on the metacarpal head. (a) Flexed metacarpophalangeal joint of a suspensory hand posture in which the joint reaction force (JRF, represented by black arrow) acts on the palmar region of the metacarpal head. Suspensory Asian apes also use hook-grips with a neutral metacarpophalangeal joint in which the JRF would act on the distal region of the metacarpal head as well; (b) Extended metacarpophalangeal joint of a knuckle-walking hand posture in which the JRF acts on the dorsal region of the metacarpal head; (c) Flexed metacarpophalangeal joint used during power grip manipulation in which JRF acts on the palmar region of the metacarpal head. Neutral metacarpophalangeal joint positions are also commonly used during manipulation in which JRF would act on the distal region of the metacarpal head.

Asian apes (*Pongo*, *Hylobates* and *Symphalangus*) most often engage in arboreal, suspensory locomotion [28], [46]. Although the comparatively slow-moving, torso-orthograde locomotion of *Pongo* differs substantially from the fast-moving, ricochetal brachiation of hylobatids, and thus magnitude of hand loading may differ, all Asian apes most commonly use a flexed-finger hand posture (i.e., hook grip or “double-locked” grip) during suspension [32], [33] (Table 1). In such a hand posture, the metacarpophalangeal joint may be in a neutral or flexed position [32], [33] with joint reaction forces (JRF) acting on the distal or palmar surfaces of the metacarpal head, respectively [23] (Fig. 1a). As such, we predict (**H1a**) that in Asian apes trabecular bone volume distribution and homogenised trabecular stiffness (i.e., region of greatest strength) will be concentrated at the distal and palmar regions of the metacarpal head epiphysis. Given the large range of motion at these joints needed for dynamic and agile suspensory or brachiating locomotion [32], we predict (**H1b**) that Asian apes will have more isotropic trabecular structure (i.e., lower degree of anisotropy) than African apes [42]. Finally, since centre of mass and gravitational forces are below the hand during suspensory locomotion, JRFs generated from forelimb compressive loads will be primarily restricted to those originating from muscle contractions [47]. As such, we predict (**H1c**) that Asian apes will have a lower trabecular bone volume fraction than African apes.

**Table 1:** Details of the study sample including primary locomotory mode.

Taxon	N	Sex (M/F/?)	Mean body mass^1^ (kg)	Relative resolution^2^ (pixels)	Primary locomotory mode	% locomotor time in primary mode
*Gorilla* 3	4	2/1/1	71.0–175.2	4.69–5.98	Knuckle-walking	∼89–98% of terrestrial locomotion^5^
*Pan paniscus*	9	5/4/0	33.2–45.0	5.33–7.94	Knuckle-walking	99.7% of terrestrial and 13–17% of arboreal locomotion^6^
*Pan troglodytes*	6	3/3/0	41.3–59.7	5.30–7.01	Knuckle-walking	32–67% of terrestrial and 11–31% of arboreal locomotion^6^
*Pongo* 4	9	2/6/1	35.6–78.5	4.35–9.32	Suspensory, torso-orthograde	35% of total locomotion^7^
*Hylobates agilis*	4	2/1/0	5.8–5.9	4.91–7.75	Suspensory, brachiator	66.3% of total locomotion^8^
*Symphalangus syndactylus*	4	0/1/3	10.7–11.9	5.20–7.35	Suspensory, brachiator	up to 80% of total locomotion^8^
*Homo sapiens*	10	5/5/0	54.4–62.2	4.42–6.48	Bipedal	hands used for manipulation and carrying

1 Sex specific mean body mass (F-M). Body masses from Smith and Jungers [49].

2 The relative resolution of the trabecular scan given here enables comparison of scan resolution relative to trabecular thickness and indicates the number of pixels representing an average trabecular strut.

3 Includes *G. gorilla* (n = 3) and *G. beringei* (n = 1).

4 Includes *P. pygmaeus* (n = 6) and *P. abelii* (n = 3).

5 [50]–[52].

6 [46], [52]–[55].

7 [28].

8 [46].

#### H2, Knuckle-walking

African apes (*Gorilla*, *Pan paniscus* and *Pan troglodytes*) most often engage in knuckle-walking locomotion (either on terrestrial or arboreal substrates; Table 1) using a hand posture in which the metacarpophalangeal joint is extended [35] (Figure 1b) and JRFs act on the dorsal surface of the metacarpal head. All African apes also engage in vertical climbing to varying frequencies (Table 1) and use hook grips with varying degrees of metacarpophalangeal joint flexion (i.e., JRFs loading the distal or palmar surfaces of the metacarpal head; [48]). Since knuckle-walking is the most frequent mode of locomotion (Table 1) in all African apes and following the results of Zeininger et al. [23], we predict that the habitual loading from JRF during climbing is less than those during knuckle-walking. As such, we predict (**H2a**) that trabecular bone volume distribution and homogenised trabecular stiffness will be more concentrated on the dorsal metacarpal head surface than the distal or palmar surface and (**H2b**) that trabecular structure will be more anisotropic (i.e. higher degree of anisotropy) than in Asian apes. Finally, since knuckle-walking creates predominantly compressive loads from JRFs arising from both contraction of muscles and gravitational forces operating on the supported body mass [47], we predict (**H2c**) that African apes will have a greater trabecular bone volume fraction than Asian apes [42].

#### H3, Manipulation

Humans (*Homo sapiens*) primarily use their hands for manipulation and typical hand postures during power grips or precision grips involve a neutral or flexed metacarpophalangeal joint posture [48], such that JRFs act on the distal or palmar regions of the metacarpal head. However, since human hands are rarely used for locomotion and body weight support, habitual joint loading is likely much lower in magnitude than that of all other apes [23], [42]. Thus we predict that human trabecular structure will be (**H3a**) relatively homogeneous throughout the metacarpal head [23], (**H3b**) trabecular bone volume will be much lower compared with all other apes [42], and (**H3c**) more isotropic than African apes [42]. If trabecular bone volume and orientation does vary, it will be concentrated in distal or palmar regions of the metacarpal head.

## Materials and Methods

### Study sample

We investigate the differences in trabecular bone parameters and distribution in the third metacarpal head of extant hominoids. The study sample is shown in Table 2. Articulated and non-articulated hand bones were loaned for study from the following institutions: Senckenburg Museum Frankfurt (*Gorilla gorilla*, *Pongo abelii*, *Pongo pygmaeus*, and *Pan troglodytes*), Berlin Museum of Natural History (*Hylobates agilis*, *Symphalangus symphalangus*, *P. abelii*, *P. pygmaeus*, *G. gorilla*, and *Gorilla beringei*), Royal Museum for Central Africa (*Pan paniscus*), Max Planck Institute for Evolutionary Anthropology (*Pan troglodytes verus*), and the Vienna Natural History Museum (*Homo sapiens*). The *Homo* sample is from an Egyptian Nubian population dated from the 6–11th centuries [56]. All non-human hominoid specimens were wild-shot. All specimens were from adult individuals and exhibited no external signs of pathology (e.g., age-related bone alteration) or trauma. Individuals were considered adult based on complete epiphyseal fusion of the external morphology throughout the hand and associated skeleton. Choice of side was dictated primarily by availability of specimens.

**Table 2:** Summary statistics for trabecular bone structure.

Taxon	N	Trabecular thickness (mm)		Scaled trabecular thickness^1^		Bone volume fraction		Degree of anisotropy^2^	
		Mean	Range	Mean	Range	Mean	Range	Mean	Range
*Gorilla*	4	0.28	0.23–0.31	0.014	0.012–0.014	0.25	0.21–0.29	0.30	0.21–0.37
*Pan troglodytes*	6	0.18	0.16–0.20	0.012	0.011–0.013	0.24	0.20–0.28	0.25	0.18–0.30
*Pan paniscus*	9	0.20	0.17–0.21	0.016	0.012–0.018	0.28	0.23–0.32	0.26	0.22–0.30
*Pongo*	9	0.22	0.15–0.27	0.016	0.012–0.021	0.20	0.13–0.32	0.18	0.01–0.34
*Symphalangus*	4	0.17	0.13–0.21	0.022	0.017–0.025	0.20	0.14–0.25	0.16	0.10–0.19
*Hylobates*	4	0.15	0.12–0.17	0.023	0.017–0.027	0.16	0.11–0.20	0.15	0.04–0.24
*Homo*	10	0.16	0.13–0.19	0.014	0.010–0.017	0.14	0.11–0.18	0.21	0.08–0.32

1 Scaled trabecular thickness is the mean trabecular thickness/geometric mean of metacarpal head size.

2 Degree of anisotropy is calculated as 1 minus the ratio of the smallest and largest eigenvalue of the fabric tensor where a DA value of 0 represents complete isotropy and a DA value of 1 represents complete anisotropy.

### Computed tomography

All specimens were scanned with a BIR ACTIS 225/300 high resolution microCT scanner at the Department of Human Evolution, Max Planck Institute for Evolutionary Anthropology (Leipzig, Germany). All specimens were scanned with an acceleration voltage of 130 kV and 100 µA using a 0.25 brass filter. Each image was reconstructed as a 2048×2048 16-bit TIFF image stack from 1250 projections with two-frame averaging. Voxel resolutions of the reconstructed scans ranged from 22–60 micrometers. Each third metacarpal was cropped from the image stack using AVIZO 6.3® (Visualization Sciences Group, SAS) and further processed separately. The Ray Casting Algorithm [57] was used to segment the bone from each image stack. Following segmentation, data were converted into 8-bit binarized images stacks in RAW format.

The scan voxel size is important for interspecific comparisons across taxa with a wide range of body sizes. Several studies have shown that certain trabecular parameters (e.g. trabecular thickness or degree of anisotropy) can be highly dependent on voxel size [58]–[60]. Therefore, we have calculated a relative resolution (mean trabecular thickness [mm]/pixel size [mm]) for each individual and taxon that represents a measure of the number of pixels assigned to an average trabecular strut [38], [58] (Table 1). Across our study sample, this value ranges from 4.35–9.32 pixels, which is equal to or higher than other similar trabecular studies [38], [58].

### Trabecular architecture analysis

Using a custom software package called ‘MedTool’ (created by DP [26]), morphological filters isolate the cortical and trabecular regions of the specimen, generate a 3D mesh of the specimen, and quantify global trabecular parameters (i.e., trabecular thickness, bone volume fraction and degree of anisotropy) throughout the entire bone or in user-defined regions of the bone (e.g., head, shaft and base). In addition to the quantified parameters, visualisation of trabecular bone volume distribution (i.e., colour map) and trabecular stiffness were produced. Gross et al. [26] provide detailed descriptions and testing of all parameters used in this study, however each step is described briefly below.

#### Segmentation of cortical and trabecular bone

**Figure 2:**
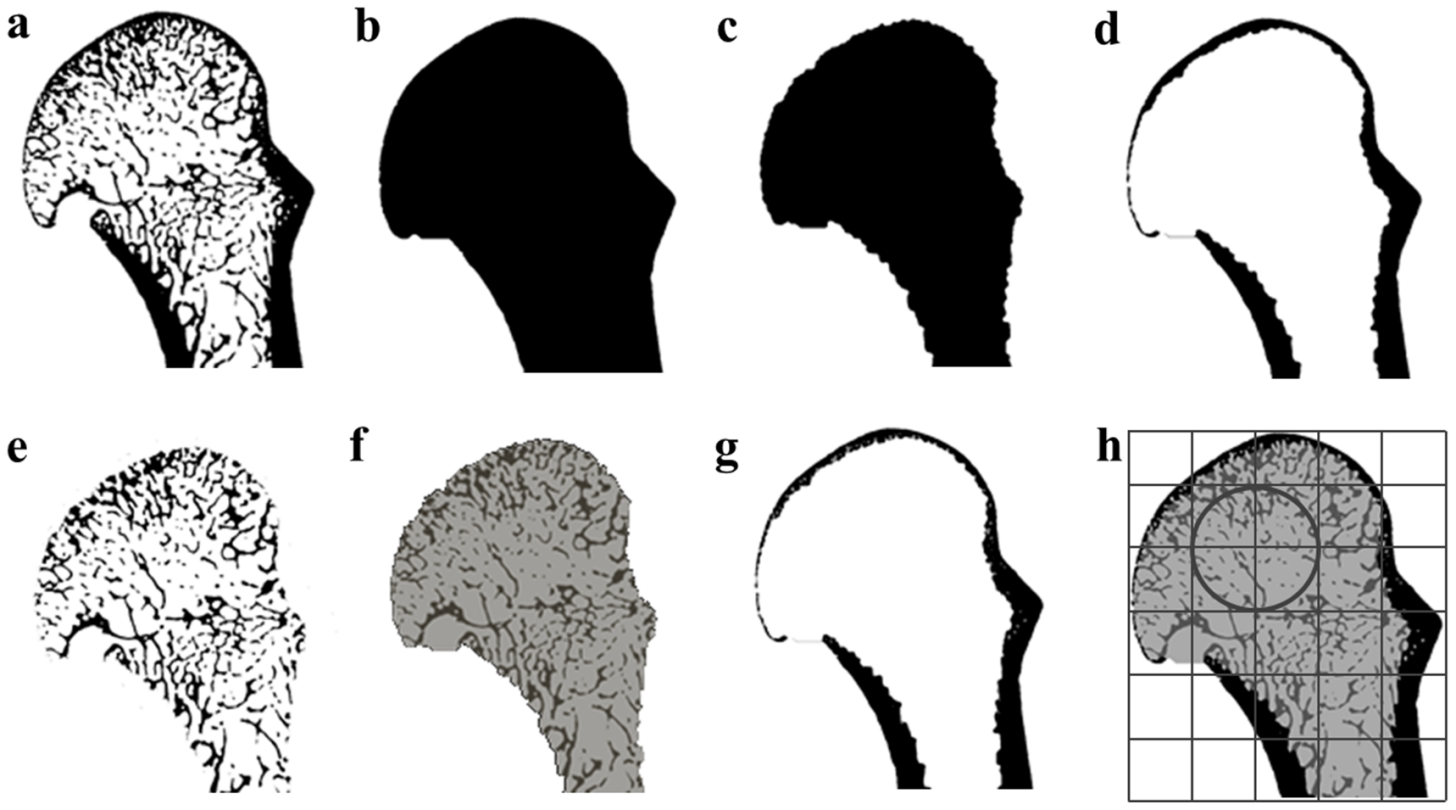
Processing stages of trabecular bone analysis. Each stage of the processing steps that separate the complete cortical and trabecular bone regions for each specimen, here shown as a sagittal cross-section in a *P. troglodytes* specimen: (a) the original segmented scan; (b) the outer surface, outlining the external surface of the cortical bone; (c) the inner surface, outlining the internal boundary between the cortical and trabecular bone; (d) the separated cortical bone model (calculated as the outer surface - inner surface); (e) the separated trabecular bone; mask images assigning different grayscale values to (f) the trabecular structure and internal “non-bone” and (g) cortex; (h) the final masked image from which trabecular structure (and cortical structure) can be quantified and the background grid and sampling sphere used to calculate bone volume fraction and the orientation of trabecular bone. Only the head region is shown here but the full bone was processed.

**Figure 3:**
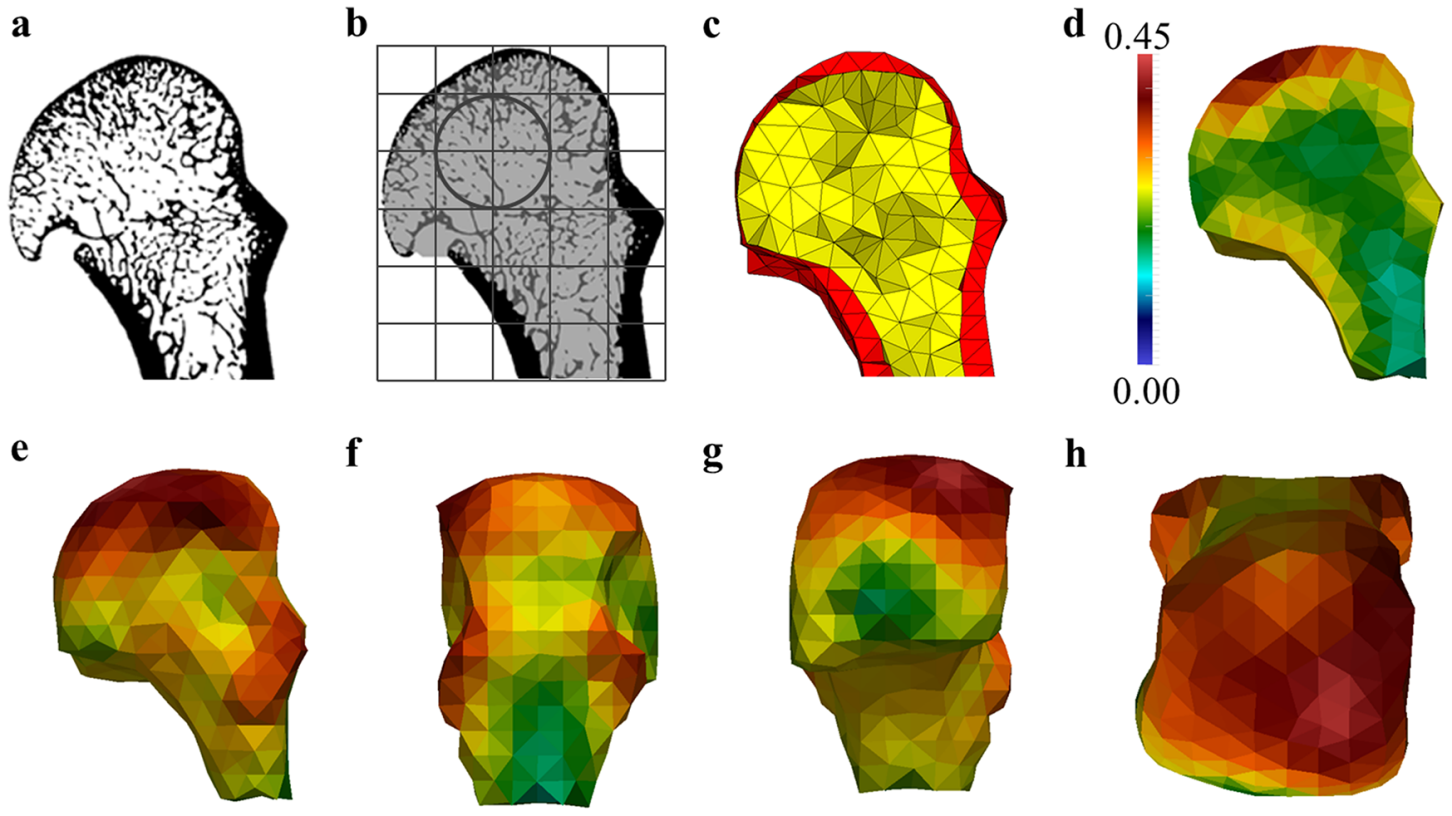
Quantifying and visualizing trabecular structure. Different stages of quantifying trabecular structure through the entire metacarpal head region, here shown as sagittal cross-sections through a *P. troglodytes* specimen as an example: (a) the original segmented scan; (b) the final masked image from which trabecular structure (and cortical structure) can be quantified and the background grid and sampling sphere used to calculate bone volume fraction and the orientation of trabecular bone (c) 3D mesh where the yellow material represents the trabecular region and the red material represents the cortex; (d) sagittal image of 3D colour map of bone volume fraction throughout the metacarpal head; (e–h) lateral, dorsal, palmar, and distal views of the 3D colour map of the bone volume fraction. The density map of the bone volume fraction is scaled from 0–0.45 BV/TV (lowest BV/TV in blue, highest BV/TV in red) in all images. Only the user-defined head region is shown here but the full bone was processed.

The cortical bone and trabecular bone were first differentiated from each other using morphological filters. Morphological closing was used to close any gaps in the cortex of segmented image (Figure 2a). Then an outer (Figure 2b) and inner (Figure 2c) surface of the bone was created. The cortex only image (Figure 2d) was created by subtracting the inner surface from the outer surface. The trabecular image (Figure 2e), was created by subtracting the cortex only image from the full image to leave only the internal region of the bone, and thus only the trabecular bone. A mask image was created that assigned a separate grey value to the cortex, trabeculae and inside (i.e., “air”) of the bone (Figure 2f–h). Next, a 2D mesh of both the inner and outer isosurfaces was generated using IsoSurf [61]–[63]. The enclosed volumes were then filled with tetrahedral finite elements using Hypermesh v11.0 (Altair), generating a 3D mesh of the trabecular and cortical region (as shown in Figure 3).

#### Measuring trabecular parameters

To isolate the metacarpal head epiphyses, the boundary of the metacarpal head was manually determined (by slice number) for each specimen. The boundary between the metacarpal head and the shaft was defined as the point where the distal end of the shaft begins to curve laterally (when viewed dorsopalmarly). Trabecular thickness (Tb.Th), bone volume fraction (BV/TV) and degree of anisotropy (DA) were calculated within this entire epiphyseal region of the bone.

The BoneJ plugin (version 1.3.1; [64]) for ImageJ (version 1.46r) was used to calculate trabecular thickness from the user-defined metacarpal head of the separated trabecular bone (Figure 2e). To quantify and visualise trabecular bone volume fraction (BV/TV) and orientation (DA), the volume is partitioned using a 2.5 mm square background grid. A sampling sphere with a 5 mm diameter is fit to each node of the grid and, within each sphere, bone volume fraction and the second rank fabric tensor (trabecular orientation) were calculated (Figure 2h and Figure 3b). These sampling dimensions are used because it includes at least five trabecular struts in each dimension, which is the smallest size that can obtain meaningful quantification of trabecular structure [26], [65]. A larger sampling sphere [66] is not appropriate given the size of hominoid metacarpal heads. For sampling spheres that protrude beyond the trabecular area, BV/TV is calculated by reducing TV (i.e., total volume) to only the volume of the sphere that is located within the trabecular region.

The fabric tensor was calculated using the mean intercept length (MIL) method [67]–[69]. The mean fabric tensor is calculated as the arithmetic mean of all second order fabric tensors, and from this the first, second and third eigenvectors and eigenvalues were extracted. The degree of anisotropy (DA) was calculated as 1 minus the ratio of the smallest and largest eigenvalue of the fabric tensor. A DA value of 0 represents complete isotropy and a DA value of 1 represents complete anisotropy.

From the biomechanical point of view, the directions of the homogenized maximum Young's moduli within the trabecular region is of particular interest. Therefore, the fourth rank stiffness tensor is calculated for each element via the Zysset-Curnier model using the in-house MedTool software [26]. The Zysset-Curnier relationship is a bone volume fraction and fabric based elasticity model that integrates local bone volume fraction and trabecular orientation in order to calculate the stiffness tensor of a material in a volume of interest [70]–[72]. The mean stiffness tensor is then calculated as the arithmetic mean of all fourth order stiffness tensors in the global coordinate system. From these stiffness tensors the direction of the maximum Young's modulus is computed.

#### Visualizing BV/TV distribution and Young's modulus

To visualize BV/TV distribution and the direction of the maximum Young's modulus (i.e., greatest stiffness) throughout the head of the third metacarpal, values calculated for these parameters at each node from the background grid were used to interpolate values at each node of the 3D mesh of the trabecular bone (see above). Interpolation of BV/TV and Young's modulus values at the nodes of the 3D mesh was done in Paraview 3.14.1 (Sandia Corporation, Kitware. Inc).

#### Measurements of the metacarpal and geometric mean

Two measurements of each metacarpal head specimen were taken on 3D surface models in Avizo 6.3: mediolateral breadth and dorsopalmar height of the metacarpal head. These measurements were used to calculate a geometric mean, which was used to scale trabecular parameters to bone size [73]. Although measures of actual body size (rarely available for museum specimens) or using a geometric mean derived from a separate skeletal element [74] may be methodologically more robust, we use a size variable derived from the metacarpal head itself in order to make the results of this study applicable to fossil specimens, for which body mass is unknown and associated skeletal elements are extremely rare.

#### Validation of methods

The robusticity of trabecular analyses ultimately depends on how accurately the trabecular structure is segmented from the original microCT scan. Thus, to test the effect of interobserver error in the Ray Casting Algorithm segmentation parameters [57], two individuals segmented the same specimen (i.e., image stack) on five separate occasions and trabecular thickness was measured again for each new segmentation. There was no significant difference in trabecular thickness measurements between individuals (Cohen's *d* = 0.10; *P* = 0.70). Validation of other aspects of the methods used in this study are available in Pahr and Zysset [65], [66] and the sensitivity of the methodological parameters are detailed in Gross et al. [26].

#### Statistical analysis

As trabecular thickness has been found to correlate with body size [44], [45], it was scaled using the geometric mean of metacarpal head size (trabecular thickness/geometric mean of metacarpal head size). Bone volume fraction and degree of anisotropy were not scaled as they shown a weak allometric relationship [45]. The Shapiro-Wilk test revealed that data were not normally distributed so non-parametric tests were used in this analysis. Due to small sample size and unknown sex of some individuals, analyses were not conducted on a sex specific basis. Non-parametric Kruskal-Wallis tests and *post hoc* pairwise comparisons were used to test for interspecific differences. Statistical tests were carried out in IBM SPSS Statistics 20, and for all tests a p-value≤0.05 was considered statistically significant.

#### Phylogenetic-integrated approach

To quantify a possible correlation between body size and trabecular parameters, regressions between Tb.Th, BV/TV and DA with (logged) body size were computed. Because our sample is interspecific, a phylogenetic-integrated approach is needed to account for similarities due to species relatedness. We use phylogenetic general least squares (PGLS) analysis with a likelihood fitted lambda model [75], [76] to quantify scaling relationships. This approach obtains estimates of regression slopes incorporating the degree of phylogenetic dependence by reference to an internal matrix of expected covariance based on the maximum likelihood estimate of lambda.

Comparative scaling approaches, however, provide only limited information about the evolutionary pathways that underlie extant diversity because they relate only to general scaling trends across the extant sample [77]. Such clade-general scaling trends therefore do not reveal how two traits have co-evolved along individual branches of the tree. It is clear that a co-evolutionary trend between two traits (expressed as a comparative correlation) is not likely expressed identically along all branches of the tree. In other words, a comparative correlation between two traits does not imply that they co-evolved to the same extent in each branch. Vice versa, the lack of a significant comparative correlation across the entire extant sample does not mean that two traits have not co-evolved along particular branches of the tree. To investigate the extent to which individual branches of the tree align with correlational trends inferred from the extant sample, we use an approach that reconstructs the evolutionary history of individual traits for each branch in the tree. This approach consists of mapping phenotypic data onto a genetically inferred phylogenetic tree. This approach allows quantifying morphological changes across time and along individual lineages of a phylogenetic tree. We hereby use the variable rates method ‘Independent Evolution’ (IE) [43] because it allows quantifying trait increase/decrease along individual lineages of the tree and has been indicated to provide independent realistic estimates of fossil primate brain and body size [43], [77]. Rates can hereby be understood as proportional changes through time. Comparing rates for specific branches between traits allows a detailed evolutionary interpretation of the processes that have shaped variation between species [77]. The phylogenetic tree was taken from the 10kTrees Project, version 3 [78], and body masses were taken from Isler et al. [79].

## Results

### Quantitative analysis of trabecular structure

Summary statistics for trabecular thickness (Tb.Th), scaled trabecular thickness, bone volume fraction (BV/TV) and degree of anisotropy (DA) are shown in Table 2. Non-parametric Kruskal-Wallis tests indicate significant differences in all trabecular parameters (Tb.Th, *p*<0.001; scaled Tb.Th, *p*<0.001; BV/TV, *p*<0.001; DA, *p* = 0.02) and the results of post-hoc pairwise comparisons for absolute and scaled trabecular thickness are shown in Table 3 and for bone volume fraction and degree of anisotropy in Table 4. Measured variables for each specimen are listed in Table S1.

**Table 3:** Kruskal-Wallis post-hoc pairwise comparisons absolute trabecular thickness (top) and scaled trabecular thickness (bottom).

Taxon	*Gorilla*	*Pan troglodytes*	*Pan paniscus*	*Pongo*	*Hylobates*	*Symphalangus*	*Homo*
*Gorilla*	-	0.009	ns	ns	**<0.001**	**0.001**	**<0.001**
*Pan troglodytes*	ns	-	ns	ns	ns	ns	ns
*Pan paniscus*	ns	0.022	-	ns	0.013	ns	0.006
*Pongo*	ns	0.008	ns	-	0.006	0.027	**0.002**
*Hylobates*	0.006	**<0.001**	0.044	ns	-	ns	ns
*Symphalangus*	0.008	**<0.001**	ns	ns	ns	-	ns
*Homo*	ns	ns	ns	ns	**0.002**	0.003	-

ns = not significant.

Bolded values remain significant after correction for multiple comparisons (adjusted significance for multiple comparisons from pairwise comparisons output of Kruskal-Wallis SPSS).

**Table 4:** Kruskal-Wallis post-hoc pairwise comparisons degree of anisotropy (top) and bone volume fraction (bottom).

Taxon	*Gorilla*	*Pan troglodytes*	*Pan paniscus*	*Pongo*	*Hylobates*	*Symphalangus*	*Homo*
*Gorilla*	-	ns	ns	0.032	0.007	0.008	0.038
*Pan troglodytes*	ns	-	ns	ns	ns	ns	ns
*Pan paniscus*	ns	ns	-	ns	0.013	0.016	ns
*Pongo*	ns	ns	0.004	-	ns	ns	ns
*Hylobates*	ns	ns	**0.002**	ns	-	ns	ns
*Symphalangus*	ns	ns	0.042	ns	ns	-	ns
*Homo*	0.005	0.004	**<0.001**	ns	ns	ns	-

ns = not significant.

Bolded values remain significant after correction for multiple comparisons (adjusted significance for multiple comparisons from pairwise comparisons output of Kruskal-Wallis SPSS).

#### Trabecular thickness (Tb.Th)

Absolute mean Tb.Th values correspond with body size differences in non-human apes; *Gorilla* and *Pongo* have the thickest trabecular bone and *Hylobates* and *Symphalangus* have the thinnest. In *Homo*, mean Tb.Th is similar to hylobatids and thus lower than expected for its body size (Tables 1 and 2). When scaled to the geometric mean of metacarpal head size, smaller taxa have relatively thicker trabeculae, indicating that the scaling may not be linear (as found by Doube et al. [44] in the femoral head across mammals). Pairwise comparisons of absolute trabecular thickness find significantly thinner trabecular bone in each hylobatid taxa than in *Gorilla* (*p*<0.001) and *Pongo* (*Hylobates p* = 0.01; *Symphalangus p* = 0.03), and in *Hylobates*, but not *Symphalangus*, when compared with *P. paniscus* (*p* = 0.01). In *Homo*, trabecular bone is significantly thinner than in other great apes, except for *Pan troglodytes* (*Gorilla p*<0.001; *P. paniscus p* = 0.01; *Pongo p*<0.01). Trabecular thickness also differs significantly between *Gorilla* and *P. troglodytes* (*p* = 0.01). After adjustment, differences in absolute trabecular thickness remain significant between *Gorilla* and the hylobatids and between *Homo* and the larger great apes: *Gorilla* and *Pongo*. Pairwise comparisons of scaled Tb.Th show significant differences between each hylobatid species and *Gorilla* (*p* = 0.01), *P. troglodytes* (*p*<0.001) and *Homo* (*p*<0.01), and between *Hylobates* and *P. paniscus* (*p* = 0.04). Within great apes, there is a significant difference in scaled trabecular thickness between *P. troglodytes* and *Pongo* (*p* = 0.01) and between the two *Pan* species (*p* = 0.02). No significant differences are found in scaled trabecular thickness within suspensory taxa. When corrected for multiple pairwise comparisons, significant differences remain between *P. troglodytes* and each hylobatid and between *Hylobates* and *Homo*.

#### Bone volume fraction (BV/TV)

*Gorilla* and *P. paniscus* have the highest mean BV/TV and *Hylobates* and *Homo* have the lowest (Table 2). *Pongo* displays high variation in BV/TV compared with all other taxa. Within the *Pongo* sample, there are no significant differences that can be attributed to sex or subspecies, however the three lowest values are from female individuals. Results of pairwise comparisons show significantly higher BV/TV in *P. paniscus* than in all suspensory taxa (*Pongo* and *Hylobates p*<0.01; *Symphalangus p* = 0.04). *Homo* has significantly lower BV/TV compared with all knuckle-walking taxa: *P. paniscus* (*p*<0.001), *P. troglodytes* (*p*<0.01) and *Gorilla* (*p*<0.01). Following correction for multiple comparisons, significant differences remain between *Pan paniscus* and two taxa: *Hylobates* and *Homo*.

#### Degree of anisotropy (DA)

**Figure 4:**
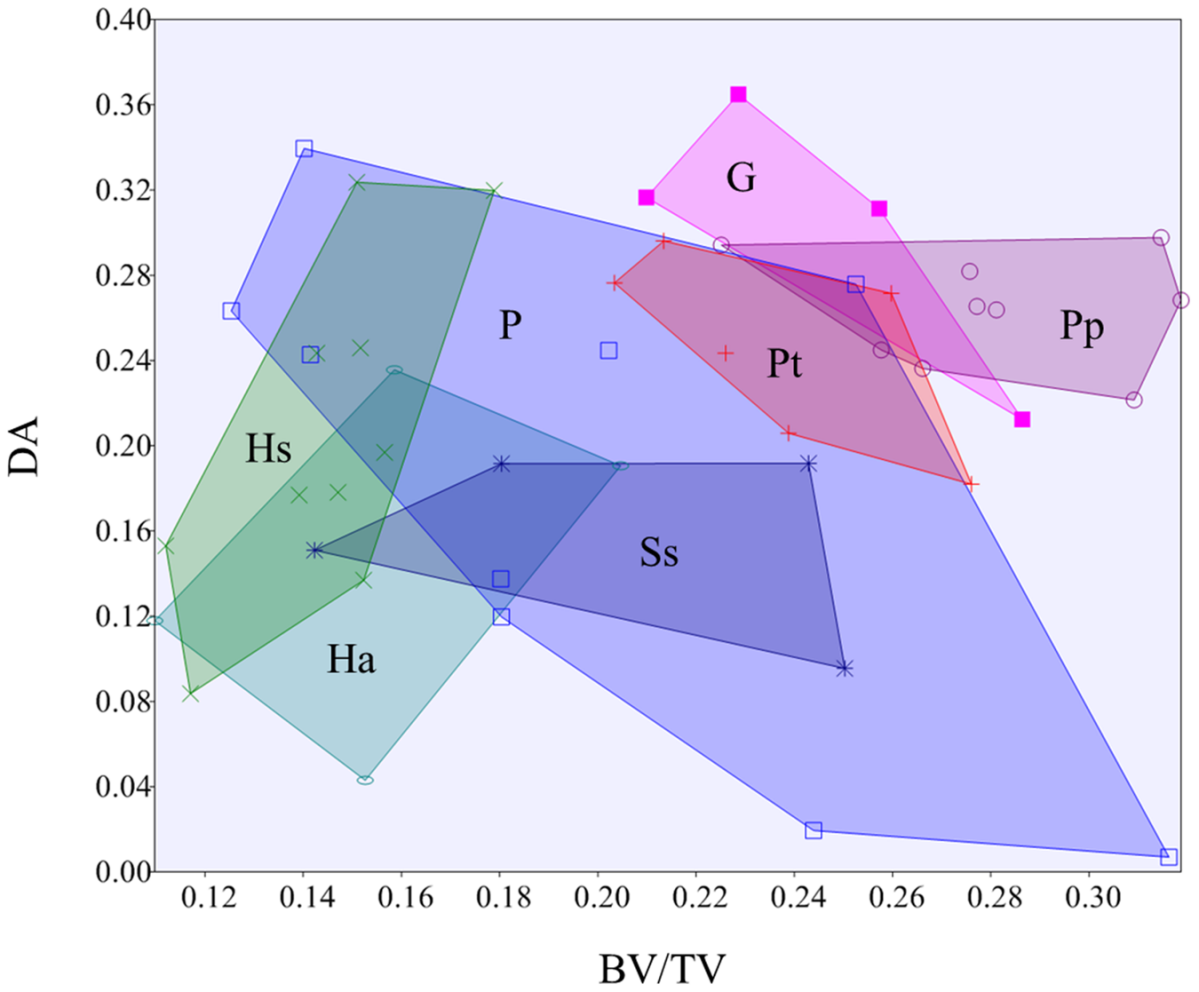
Bivariate plot of bone volume fraction (BV/TV) and degree of anisotropy (DA). Knuckle-walking taxa (*Gorilla* and *Pan*) are characterised by a high BV/TV and, to a lesser extent, high DA in comparison with the brachiating taxa (*Hylobates* and *Symphalangus*). *Pongo* is highly variable in both BV/TV and DA. *Homo* has a comparatively low BV/TV and a large range of DA values. (**G** *Gorilla*; **Pp** *P. paniscus*; **Pt** *P. troglodytes*; **P** *Pongo*; **Ss** *Symphalangus syndactylus*; **Hs** *Homo sapiens*; **Ha** *Hylobates agilis*).

*Gorilla* and *P. paniscus* have the highest DA, *Hylobates* and *Symphalangus* have the lowest, and *Pongo* shows a large range of variation. Pairwise comparisons reveal significant differences between *Gorilla* and *Hylobates* (*p* = 0.01), *Symphalangus* (*p* = 0.01), *Pongo* (*p* = 0.03), *Homo* (*p* = 0.04), and between *P. paniscus* and *Hylobates* (*p* = 0.01), and *Symphalangus* (*p* = 0.02). However, none of these pairwise comparisons retain their significance after correction for multiple comparisons. Figure 4 presents a bi-variate plot of variation in BV/TV and DA across the study taxa. Knuckle-walking taxa have a higher BV/TV and DA than the brachiating species. However, there is a high degree of interspecific variation in the results for *Pongo* (and no correlation was found between BV/TV and DA and sex or subspecies).

### Phylogenetic reconstruction of changes in trabecular structure

**Figure 5:**
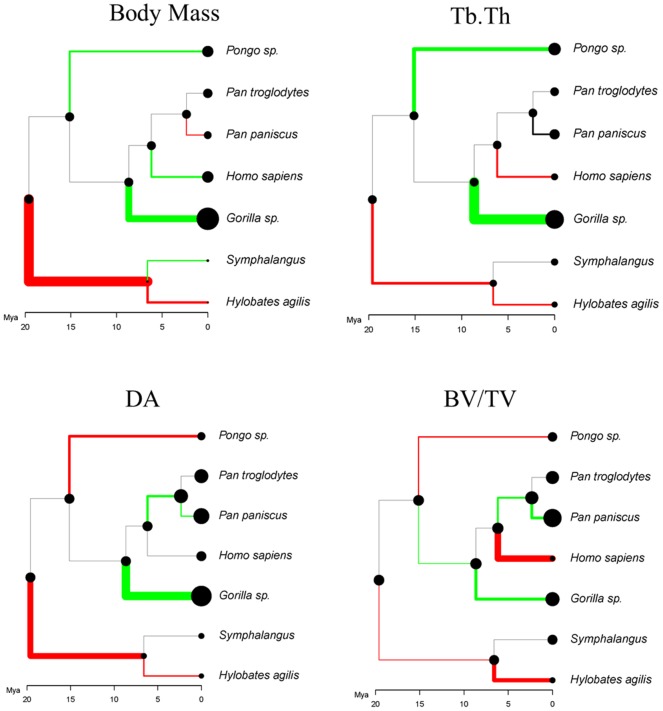
Phylogenetic reconstructions of evolutionary changes in body mass and trabecular bone parameters. Taking into account the divergence dates of the hominoid linage, these evolutionary reconstructions show how body mass, trabecular thickness (Tb.Th), bone volume fraction (BV/TV), and degree of anisotropy (DA) are estimated to have changed over time based on the trabecular structure present in extant hominoid metacarpals. The x axis represents time in millions of years (Mya). Green represents an increase and red a decrease, the thickness of the line reveals the rate of change.

Taking into account the divergence dates of the hominoid lineage, the evolutionary reconstructions shown in Figure 5 show how body mass and three trabecular parameters – Tb.Th, BV/TV and DA - are estimated to have changed since the last common ancestor. Green represents an increase and red a decrease, the thickness of the line reveals the rate of change. In *Hylobates* and *Symphalangus* – the brachiating lineage – there is a decrease in all three trabecular parameters: between the last common ancestor of hominoids and the last common ancestor of the hylobatids, the greatest reduction is in DA, then Tb.Th and then BV/TV. *Hylobates* shows further reduction in all trabecular parameters, distinct from *Symphalangus*. In the ancestral *Hylobates* lineage, reduction in body size exceeds that of trabecular parameters in all branches, except for BV/TV and, to a lesser extent, Tb.Th. Between the last common ancestor of great apes and extant *Pongo*, there is an increase in Tb.Th, a reduction in DA and, to a lesser extent, a reduction in BV/TV compared with an increase in body mass. Knuckle-walking taxa show different evolutionary patterns; in the *Gorilla* lineage, there is an increase in Tb.Th and DA comparable to the increase in body mass, whereas BV/TV increases less than body mass increases. After divergence from the hominin lineage, the *Pan* lineage also shows an increase, albeit much smaller than *Gorilla*, in BV/TV and DA, but there is only minimal change in trabecular thickness and body mass. *P. paniscus* further diverges from *P. troglodytes* with an increase in both BV/TV and, to a lesser extent, DA despite a reduction in body mass. In contrast to all other hominines, *Homo* shows a reduction (rather than increase) in Tb.Th and, especially, BV/TV in contrast to the increase in body mass since its shared common ancestor with *Pan*. Phylogenetic least squares regression found no significant correlations between logged body size and any trabecular parameter. *Homo* deviates from the general pattern in trabecular thickness, and regressions excluding *Homo* were significant for Tb.Th (*p*<0.03, λ = 0.67, R^2^ = 0.73), but not for BV/TV or DA.

### Qualitative analysis of trabecular structure

**Figure 6:**
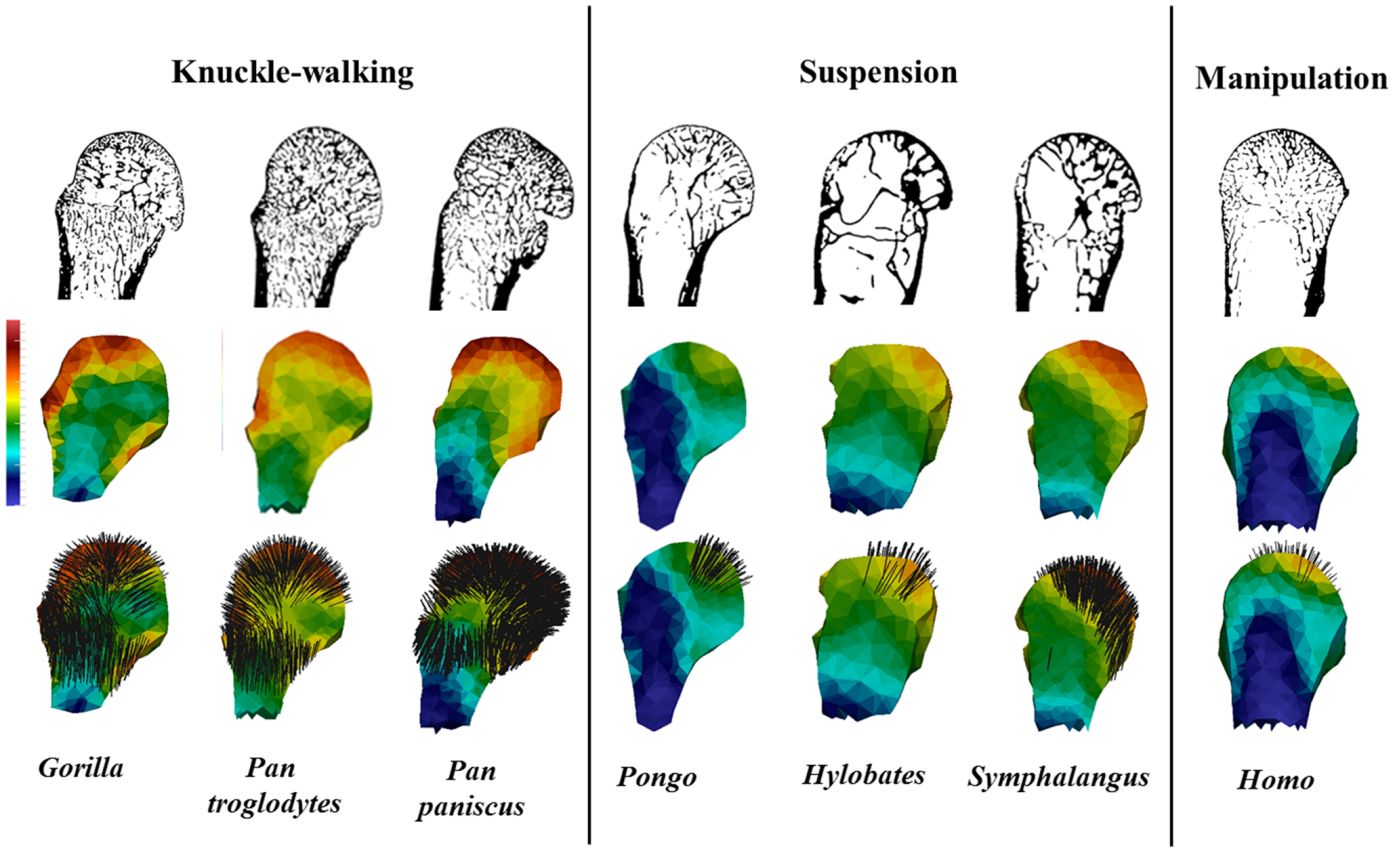
Trabecular structure in the hominoid third metacarpal head. Sagittal midline cross-section of the third metacarpal head in three views. Top row, a cross-section of the original segmented scan, showing variation in trabecular structure across taxa. Middle row, a colour map of bone volume (BV/TV) distribution scaled to 0–0.45 (cortical bone has been removed). Bottom row, directions of greatest stiffness superimposed on the bone volume colour map. Only regions with high Young's moduli (>1000 Pa) are considered in all taxa.

Figure 6 presents images of one representative third metacarpal head from each taxon in three sagittal midline cross-sections: (1) the actual trabecular structure, (2) colour map of the trabecular bone volume distribution and, (3) areas and directions of greatest trabecular stiffness (taking into consideration trabecular orientation and BV/TV to represent Young's modulus). These images are available for the full sample of each taxon in Figures S1, S2, S3, S4, S5, S6, S7.

#### Trabecular structure

Sagittal midline cross-sections of the metacarpal head reveal differences in trabecular structure across taxa (Figure 6, top row). The metacarpal head of knuckle-walking apes has a dense trabecular structure compared with brachiators, which have relatively few, but long trabeculae with few connections. *Pongo* is highly variable; some individuals have relatively sparse trabecular structure and some relatively dense (Figure S4). *Homo* appears intermediate between the two groups, with a trabecular structure that is less dense than in the knuckle-walking apes and *Pongo* but more numerous than the brachiators. Knuckle-walking apes appear to have a more homogenous distribution of trabeculae throughout the metacarpal head whereas in all suspensory taxa the trabeculae are concentrated in the palmar and distal portions of the head. Within the knuckle-walkers, both *Pan* species appear to have more numerous, highly connected trabecular structure than that of *Gorilla*.

#### Trabecular bone volume distribution

A colour map reflecting the distribution of trabecular bone volume (i.e., the cortical shell has been removed), confirms the initial observations described above based on the trabecular structure (Figure 6, middle row). Across all individuals, regions of highest bone volume are restricted to just below the articular surface. Knuckle-walking apes have a more homogeneous bone volume distribution that reaches the dorsal region of the metacarpal head. In contrast, in suspensory apes the highest regions of bone volume are localised to the distopalmar regions of the articular surface. *Homo* generally shows a similar pattern to that of suspensory apes, with a slightly more distal localisation to the overall trabecular volume. Within knuckle-walking apes, *Gorilla* shows a stronger dorsal concentration and lower palmar concentration of trabeculae, while *Pan* has a more consistent distribution of trabecular bone throughout the articular surface of metacarpal head. Comparisons among the three suspensory species reveal that *Pongo* has a more concentrated palmar distribution of trabeculae than that of brachiators. Although *Pongo* has more numerous trabeculae, the brachiators have relatively thicker trabeculae (Table 2), which explains their higher bone density in the colour maps.

#### Homogenised trabecular stiffness

Figure 6 (bottom row) presents the direction of the highest stiffness superimposed on the bone volume distribution colour map described above. In this image, only regions with a high homogenised stiffness (Young's modulus above 1000 Pa) are considered in order to reveal the stiffest regions of the bone and to compare regions across taxa that have the same homogenised elastic behaviour. The direction of the lines indicates the direction of the highest stiffness (principal direction of the stiffness tensor) at each background grid node. All knuckle-walking taxa demonstrate a greater proportion of the metacarpal head with homogenised trabecular stiffness above the threshold value than suspensory taxa. *Homo* is the least stiff compared with all other apes. In knuckle-walking apes, stiffness is high throughout most of the articular region, reaching to the dorsum. Of particular interest is the high stiffness concentration under the dorsal ridge (labelled in Figure 1). When the Young's modulus threshold is raised to 2000 Pa, knuckle-walking apes show the highest stiffness at the distopalmar surface and in most, but not all, individuals on the dorsal ridge. In contrast, in suspensory apes, the highest stiffness is localised to the palmar articular region. Throughout the sample, only a single *Hylobates* and a single *Homo* individual have no stiffness values above the Young's modulus of 1000 Pa threshold. In *Homo*, the stiffest region is more distally oriented than in suspensory apes (Figure S7).

## Discussion

Although much experimental research has demonstrated the response of trabecular bone to mechanical stress throughout life [2]–[5], previous studies of trabecular bone structure in primate epiphyses – especially the humeral and femoral head – have often failed to identify clear differences in joint loading associated with habitual behaviours (e.g. [11], [16], [21], [25]). The ambiguous results of some previous studies may be partly due to application of traditional volume of interest-based methods to complex and more proximal joints that are further removed from substrate reaction forces than more distal anatomical regions. This study applied a novel method to quantify trabecular structure in the third metacarpal head, an anatomical region that more directly incurs substrate (or tool) reaction forces during hand use. The goal of this study was to determine whether variation in trabecular structure throughout the metacarpal head reflects peak joint loading during habitual hand postures associated with different locomotor and manipulative behaviours across extant apes. We tested three hypotheses related to primary mode of hand use, hand posture and loading: (1) suspensory locomotion using a flexed or neutral metacarpophalangeal joint and low compressive loading, (2) knuckle-walking locomotion using an extended metacarpophalangeal joint and high compressive loading and (3) manipulation using a flexed or neutral metacarpophalangeal joint and a low overall magnitude of loading.

### Is there a behavioural signal in the third metacarpal head?

This study found qualitative and quantitative differences in trabecular structure across hominoids that reflect predicted variation in metacarpophalangeal joint peak loading and hand posture, and generally support our hypotheses.

#### Suspension

We predicted that suspensory Asian apes would show (**H1a**) a distopalmar concentration in highest trabecular bone volume and homogenised trabecular stiffness (i.e., greatest stiffness) and, compared with African apes, (**H1b**) a more isotropic trabecular structure and (**H1c**) lower BV/TV. All of these hypotheses were supported, although *Pongo* demonstrated a large range of variation in trabecular structure such that some individuals did not differ quantitatively from that of African apes. Results from qualitative 3D visualization of trabecular structure demonstrated that trabecular bone volume was concentrated on the palmar surface in suspensory hominoids, corresponding with a flexed metacarpophalangeal joint grip commonly used in suspensory locomotion (**H1a**) [32], [33]. The higher stiffness values along the more distal articular region in hylobatids may reflect higher loading and more frequent use of hook grips than in *Pongo*. Quantitatively, suspensory taxa generally had more isotropic (i.e. lower DA values) and lower BV/TV than knuckle-walking taxa, as predicted. This pattern was true for all hylobatids, but did not hold across all *Pongo* individuals, given the large range of variation in its trabecular structure.

*Pongo* showed a much larger degree of variability in trabecular structure (Tb.Th, BV/TV and DA) compared with all other taxa in our study sample. This result was also found in a previous study of trabecular structure in hominoid carpal bones from the same study sample [80], [81]. This variation may reflect more terrestrial behaviour (i.e., fist-walking) by some individuals compared with others [82], [83], although we found no correlation in our sample between trabecular structure and sex (males tend to be more terrestrial than females) or species (*P. pygmaeus* is more terrestrial than *P. abelii*). Future research should assess whether this pattern of variation is systemic within and between individuals, given that similar variability was not reported in the humeral or femoral head [24], [25], vertebrae [18], or metatarsals [19].

#### Knuckle-walking

Our predictions that knuckle-walking African apes would show (**H2a**) a dorsal concentration in trabecular bone volume and homogenised trabecular stiffness, and compared with all other taxa, (**H2b**) more anisotropic trabecular structure and (**H2c**) greater BV/TV, were supported. Qualitative visualization of the trabecular structure revealed that a high density of trabecular bone and high trabecular stiffness throughout the metacarpal head, but particularly the dorsal regions and underneath the dorsal ridge, which bears a striking contrast to the pattern seen in suspensory taxa and *Homo* (see below). As dorsal ridges are thought to limit hyperextension of the metacarpophalangeal joint during knuckle-walking [84], and have been considered an adaptive response to loading during life [85], [86], it is not surprising that the trabecular bone in this region may also be responding to loading. The stiffness orientations in all knuckle-walking African apes are oriented dorsally and radiate around the entire articular surface, particularly in *P. paniscus*. This pattern may reflect the additional arboreal behaviours in which all African apes engage, and *P. paniscus* in particular [55]. High trabecular volume and directionality was confirmed quantitatively, revealing that knuckle-walking taxa generally had higher BV/TV and slightly higher DA values than hylobatids, *Homo* and some *Pongo* individuals.

Within African apes, species vary in their frequency of knuckle-walking and its associated ecological context; *G. beringei* engages almost solely in terrestrial knuckle-walking [52], [55] while *P. paniscus* and *P. troglodytes* engage in more arboreal knuckle-walking and climbing [51], [53]–[55], [87], [88]. It might be expected that as the most arboreal of the knuckle-walking taxa, *P. paniscus* [54], would have a trabecular structure intermediate between African apes and Asian apes, consistent with findings for other features of the metacarpal morphology, such as cortical bone thickness [84]. However, this study found a higher (rather than lower) BV/TV and more even distribution of trabecular bone in *P. paniscus* compared with the other knuckle-walking species. This pattern is consistent with previous studies on the first metacarpal [89] and first and second metatarsal head [19] in which *P. paniscus* had higher BV/TV than *P. troglodytes* and *Gorilla*. Further investigation of variation in locomotor behaviours and hand (and foot) use among African apes or genetic influences affecting trabecular bone density throughout the skeleton would help clarify these interspecific differences.

#### Manipulation

Finally, we predicted that since humans most often use their hands for manipulation, in which loads are likely much lower than those incurred during locomotion and weight-bearing, *Homo* would demonstrate (**H3a**) a homogeneous trabecular structure throughout the metacarpal head, (**H3b**) a lower BV/TV compared with all other taxa and (**H3c**) more isotropic trabecular structure than knuckle-walking taxa. Again, these hypotheses were generally supported. Qualitative depictions of the trabecular structure revealed that *Homo* has relatively little trabecular structure that is more homogeneous than suspensory taxa. However, there was a higher concentration and stiffness along the distal region of the articular surface, which is consistent with peak loading in a neutral metacarpophalangeal joint position. As predicted, *Homo* demonstrated much lower BV/TV than all other taxa and lower mean DA than knuckle-walking apes.

Our results suggest that a higher BV/TV and, to a lesser extent, higher DA are associated with quadrupedal locomotion compared with non-quadrupedal behaviours in the metacarpal head. Higher BV/TV is considered important for resisting higher compression in quadrupedal locomotion compared with suspensory locomotion [47]. A higher degree of anisotropy has been suggested to be caused by more predictable loading regimes during locomotion in the femoral head of leaping strepsirrhine taxa compared with slow-moving quadrupedal strepsirrhines [11], [13]. Yet previous studies have found inconsistent results for the correlation between quadrupedalism and a higher BV/TV compared with other locomotor modes (e.g., suspension, leaping, bipedalism) [11], [16], [19], [21], [24], [25]. Similarly, analyses of the humeral and femoral head across several anthropoid taxa did not find a consistent link between DA and the predictability of loading [16], [18], [24]. However, in the foot, which, like the hand, is in direct contact with the locomotor substrate, a higher degree of anisotropy has been found in *Homo* compared with those of non-human apes (small sample of calcanei [41]; metatarsals [19]), associated with more stereotypical loading in the bipedal gait than in other non-human apes where the foot is used for climbing and manipulation.

The whole-epiphysis methodological approach used here reveals variation in trabecular structure across the hominoid third metacarpal head that correlates with predicted joint position and peak loading during hand use. Thus, this method offers great potential for analyses of fossil taxa that preserve internal bony structure and the reconstruction of hand use in the past, as well as other anatomical areas in extant and fossil taxa. It is important to note that functional signals in relative bone volume and stiffness found in this study were primarily located adjacent to the articular surface, where joint reaction forces are first incurred and trabecular bone is more likely to respond and remodel. Our results are consistent with those of Zeininger et al. [23], which found similar functional signals in the *Pan*, *Pongo*, and *Homo* metacarpal head using backscatter electron image analysis of subchondral and trabecular bone mineral density. Volume of interest-based methods typically quantify trabecular structure in a central region of the epiphyses (as to avoid any inclusion of cortex) and thus these functional signals in trabecular bone can be missed. This may explain the absence of clear behavioural signals in previous trabecular studies [16], [21], [24], [25].

Although our analysis has revealed clear differences in trabecular structure associated with predicted hand posture and peak loading of the metacarpal head, it should be acknowledged that not all of the variation in trabecular structure may be explained by joint function. This study found that *Homo* had particularly low BV/TV and, conversely, *Pan* had particularly high BV/TV compared with the other taxa. These disparate patterns are also found in other areas of the skeleton in these taxa. Compared with several other anthropoid primates, *Homo* has much lower BV/TV in the humeral and femoral head [24] and, compared with other great apes, low BV/TV in the calcaneus [41] and metatarsal heads [19], despite high loading from bipedalism and toe-off. These same studies revealed extremely high BV/TV in *Pan* humeral and femoral head [24] and first metatarsal head [19]. The consistency of these results throughout regions of the skeleton that incur different loading suggests there may be a strong systemic influence associated with trabecular structure in at least some taxa that requires further investigation [2], [90].

Phylogenetic reconstruction of trabecular parameters in each lineage reveals divergent changes in trabecular parameters, some of which may be associated with species specific trabecular patterning. Evolutionary changes in trabecular parameters differ between knuckle-walking taxa. In both *Gorilla* and *Pan*, DA and BV/TV have increased, however in *Pan* this is not associated with an increase in body size or Tb.Th. The increase in DA and BV/TV in knuckle-walkers may reflect locomotor loading in knuckle-walking, as predicted (**H2a, H2b**). In *Homo*, the reduction in Tb.Th and BV/TV compared with the increase in body size supports our prediction (**H3b**) that the trabecular structure in this species is characterised by a reduction in joint loading associated with removal of the forelimb from locomotion. Among suspensory taxa, there has been a reduction in DA and, to a lesser extent, BV/TV which corresponds with our predictions (**H1b, H1c**) due to reduced compressive loading in suspensory species, and a greater range of joint motion. Changes in Tb.Th in suspensory taxa may be due to divergence in body size, as trabecular thickness was found to correlate with body size in non-human hominoids. Based on phylogenetic comparisons it does not seem that phylogenetic relatedness was responsible for differences in trabecular bone patterning in the metacarpal head. It therefore appears that changes in trabecular parameters in the hominoid lineage may be due to species specific trabecular patterning or to functional loading in locomotor and manipulatory behaviour. Our results indicating a lack of a significant scaling relationship between estimated body mass and trabecular parameters in the PGLS analysis, are inconsistent with our results derived from regressions of metacarpal head size (as a proxy for body mass) and these same trabecular parameters. In both cases our analysis suffers from poor sample sizes and an inability to adequately assess scaling intraspecifically. Therefore, we would suggest that future analyses should attempt to comprehensively explore scaling relationships of trabecular structure in hominoids taking into account intraspecific variation and phylogeny (although we are all too aware of the methodological difficulties in acquiring appropriate data on individual-specific body size and large samples of microtomographic scans).

In this study the whole-epiphysis method revealed variation in the trabecular structure of the third metacarpal head corresponding with functional loading during knuckle-walking, suspensory and manipulative behaviour. This method has potential for resolving longstanding debates interpreting both locomotor and manipulative behaviour in fossil hominoids and hominins.

## Supporting Information

Table S1 Complete study sample with raw data for each trabecular variable.
(DOCX)

Figure S1 ***Gorilla*** **sample, shown in a sagittal midline cross-sections of the third metacarpal head in four views.** From left to right: cross section of the original scan; colour map of bone volume (BV/TV) distribution scaled to 0–0.45 (cortical bone has been removed); stiffness tensor maximum orientations superimposed on the bone volume colour map, the stiffness tensor is thresholded to E-modulus = 1000 Pa. Dorsal view of stiffness tensor maximum orientations superimposed on the bone volume colour map.
(TIF)

Figure S2 ***Pan troglodytes*** **third metacarpal head sample, shown in same views as described in Figure S1.**
(TIF)

Figure S3 ***Pan paniscus*** **third metacarpal head sample, shown in same views as described in Figure S1.**
(TIF)

Figure S4 ***Pongo*** **third metacarpal head sample, shown in same views as described in Figure S1.**
(TIF)

Figure S5 ***Hylobates agilis*** **third metacarpal head sample, shown in same views as described in Figure S1.**
(TIF)

Figure S6 ***Symphalangus syndactylus*** **third metacarpal head sample, shown in same views as described in Figure S1.**
(TIF)

Figure S7 ***Homo sapiens*** **third metacarpal head sample, shown in same views as described in Figure S1.**
(TIF)

## References

[1] Ward C (2002) Interpreting the posture and locomotion of *Australopithecus afarensis*: where do we stand? Yrbk Phys Anthropol 35: 185–215. 10.1002/ajpa.10185 12653313

[2] Currey JD (2002) Bones: structure and mechanics. Princeton: Princeton University Press.

[3] Pontzer H, Lieberman DE, Momin E, Devlin MJ, Polk JD, et al (2006) Trabecular bone in the bird knee responds to high sensitivity to changes in load orientation. J Exp Biol 209: 57–65. 16354778 10.1242/jeb.01971

[4] Ruff C, Holt B, Trinkaus E (2006) Who's afraid of the big bad Wolff?: “Wolff's law” and bone functional adaptation. Am J Phys Anthropol 129: 484–498. 16425178 10.1002/ajpa.20371

[5] Barak MM, Lieberman DE, Hublin J-J (2011) A Wolff in sheep's clothing: Trabecular bone adaptation in response to changes in joint loading orientation. Bone 49: 1141–1151. 21893221 10.1016/j.bone.2011.08.020

[6] Ruff CB, Runestad JA (1992) Primate limb bone structural adaptations. Annu Rev Anthropol 21: 407–433.

[7] Wolff J (1892) Das Gesetz der Transformation der Knochen. Berlin: A. Hirchwild.

[8] Pearson OM, Lieberman DE (2004) The aging of Wolff's “law”: ontogeny and responses to mechanical loading in cortical bone. Yrbk Phys Anthropol 47: 63–99. 10.1002/ajpa.20155 15605390

[9] Martin RB, Burr DB, Sharkey NA (2010) Skeletal Tissue Mechanics. New York: Springer-Verlag.

[10] Fajardo RJ, Müller R (2001) Three-dimensional analysis of nonhuman primate trabecular architecture using micro-computed tomography. Am J Phys Anthropol 115: 327–336. 11471131 10.1002/ajpa.1089

[11] Ryan TM, Ketcham RA (2002) The three-dimensional structure of trabecular bone in the femoral head of strepsirrhine primates. J Hum Evol 43: 1–26. 12098207 10.1006/jhev.2002.0552

[12] Ryan TM, Ketcham RA (2002) Femoral head trabecular structure in two omomyid primates. J Hum Evol 43: 241–263. 12160718 10.1006/jhev.2002.0575

[13] MacLatchy L, Müller R (2002) A comparison of the femoral head and neck trabecular architecture of *Galago* and *Perodicticus* using micro-computed tomography (mCT). J Hum Evol 43: 89–105. 12098212 10.1006/jhev.2002.0559

[14] Ryan TM, Ketcham RA (2005) Angular orientation of trabecular bone in the femoral head and its relationship to hip joint loads in leaping primates. J Morphol 265: 249–263. 15690365 10.1002/jmor.10315

[15] Ryan TM, van Rietbergen B (2005) Mechanical significance of femoral head trabecular bone structure in *Loris* and *Galago* evaluated using micromechanical finite element models. Am J Phys Anthropol 126: 82–96. 15386240 10.1002/ajpa.10414

[16] Fajardo RJ, Müller R, Ketcham RA, Colbert M (2007) Nonhuman anthropoid primate femoral neck trabecular architecture and its relationship to locomotor mode. Anat Rec 290: 422–436. 10.1002/ar.20493 17514766

[17] Lazenby RA, Cooper DML, Angus S, Hallgrímsson B (2008) Articular constraint, handedness, and directional asymmetry in the human second metacarpal. J Hum Evol 54: 875–885. 18207490 10.1016/j.jhevol.2007.12.001

[18] Cotter M, Simpson S, Latimer B, Hernandez C (2009) Trabecular microarchitecture of hominoid thoracic vertebrae. Anat Rec 292: 1098–1106. 10.1002/ar.20932 19554642

[19] Griffin NL, D'Août K, Ryan TA, Richmond BG, Ketcham RA, et al (2010) Comparative forefoot trabecular bone architecture in extant hominids. J Hum Evol 59: 202–213. 20655571 10.1016/j.jhevol.2010.06.006

[20] Macho GA, Spears IR, Leakey MG, McColl DJ, Jiang Y, et al (2010) An exploratory study on the combined effects of external and internal morphology on load dissipation in primate capitates: its potential for an understanding of the positional and locomotor repertoire of early hominins. Folia Primatol 81: 292–304. 21242695 10.1159/000322631

[21] Ryan TM, Walker A (2010) Trabecular bone structure in the humeral and femoral heads of anthropoid primates. Anat Rec 293: 719–729. 10.1002/ar.21139 20235327

[22] Lazenby RA, Skinner MM, Hublin J-J, Boesch C (2011) Metacarpal trabecular architecture in the chimpanzee (*Pan troglodytes*): evidence for locomotion and tool use. Am J Phys Anthropol 144: 215–225. 20872805 10.1002/ajpa.21390

[23] Zeininger A, Richmond BG, Hartman G (2011) Metacarpal head biomechanics: A comparative backscattered electron image analysis of trabecular bone mineral density in *Pan troglodytes*, *Pongo pygmaeus*, and *Homo sapiens* . J Hum Evol 60: 703–710. 21316735 10.1016/j.jhevol.2011.01.002

[24] Ryan TM, Shaw CN (2012) Unique suites of trabecular bone features characterize locomotor behavior in human and non-human anthropoid primates. PLoS ONE 7 7: e41037 doi:10.1371/journal.pone.0041037 22815902 10.1371/journal.pone.0041037 PMC3399801

[25] Shaw CN, Ryan TM (2012) Does skeletal anatomy reflect adaptation to locomotor patterns? Cortical and trabecular architecture in human and nonhuman anthropoids. Am J Phys Anthropol 147: 187–204. 22120605 10.1002/ajpa.21635

[26] Gross T, Kivell TL, Skinner MM, Huynh Nguyen N, Pahr DH (in review) A CT-image-based method for the holistic analysis of cortical and trabecular bone. Submitted to Pal Elec

[27] Cant JGH (1987) Positional behavior of female Bornean orangutans (*Pongo pygmaeus*). Am J Primatol 12: 71–90. 10.1002/ajp.1350120104 31973515

[28] Thorpe SKS, Crompton RH (2006) Orangutan positional behavior and the nature of arboreal locomotion in Hominoidea. Am J Phys Anthropol 131: 384–401. 16617429 10.1002/ajpa.20422

[29] Fleagle JG (1974) Dynamics of a brachiating siamang *Hylobates* (*Symphalangus*) *syndactylus* . Nature 248: 259–260. 4819422 10.1038/248259a0

[30] Fleagle JG (1976) Locomotion and posture of the Malayan siamang and implications for hominoid evolution. Folia Primatol 26: 245–269. 1010498 10.1159/000155756

[31] Hollihn U (1984) Bimanual suspensory behavior: morphology, selective advantages and phylogeny. In: Preuschoft H, Chivers DJ, Brokelman WY, Creel N, editors. The lesser apes: Evolutionary and behavioral biology. Edinburgh: Edinburgh University Press. pp. 85–95.

[32] Rose M (1988) Functional anatomy of the Cheiridia. In: Schwartz J, editor. Orang-utan biology. Oxford:Oxford University Press. pp. 299–310.

[33] Richmond BG (2007) Biomechanics of phalangeal curvature. J Hum Evol 53: 678–690. 17761213 10.1016/j.jhevol.2007.05.011

[34] Tuttle RH (1967) Knuckle-walking and the evolution of hominoid hands. Am J Phys Anthropol 26: 171–206.

[35] Jenkins FA, Fleagle JG (1975) Knuckle-walking and the functional anatomy of the wrist. In: Tuttle CH, editor. Primate functional morphology and evolution. The Hague: Mouton. pp. 213–227.

[36] Wunderlich RE, Jungers WL (2009) Manual digital pressures during knuckle-walking in chimpanzees (*Pan troglodytes*). Am J Phys Anthropol 139: 394–403. 19170201 10.1002/ajpa.20994

[37] Jones LA, Lederman SJ (2006) Human hand function. Oxford: Oxford University Press.

[38] Kivell TL, Skinner MM, Lazenby R, Hublin J-J (2011) Methodological considerations for analyzing trabecular architecture: an example from the primate hand. J Anat 218: 209–225. 20977475 10.1111/j.1469-7580.2010.01314.x PMC3042755

[39] Lazenby RA, Skinner MM, Kivell TL, Hublin J-J (2011) Scaling VOI size in 3D μCT studies of trabecular bone: a test of the oversampling hypothesis. Am J Phys Anthropol 144: 196–203. 20979207 10.1002/ajpa.21385

[40] Skedros JG, Baucom SL (2007) Mathematical analysis of trabecular ‘trajectories’ in apparent trajectorial structures: the unfortunate historical emphasis on the human proximal femur. J Theor Biol 244: 15–45. 16949618 10.1016/j.jtbi.2006.06.029

[41] Maga M, Kappelman J, Ryan T, Ketcham RA (2006) Preliminary observations on the calcaneal trabecular microarchitecture of extant large-bodied hominoids. Am J Phys Anthropol 129: 410–417. 16323186 10.1002/ajpa.20276

[42] Zylstra M (2000) Trabecular architecture of metacarpal heads in catarrhines: a preliminary report. Am J Phys Anthropol Supp 30: 331.

[43] Smaers JB, Vinicius L (2009) Inferring macro-evolutionary patterns using an adaptive peak model of evolution. Evol Ecol Res 11: 991–1015.

[44] Doube M, Kłosowski MM, Wiktorowicz-Conroy AM, Hutchinson JR, Shefelbine SJ (2011) Trabecular bone scales allometrically in mammals and birds. Proc R Soc Lond B Biol Sci 278: 3067–3073. 10.1098/rspb.2011.0069 PMC3158937 21389033

[45] Ryan TM, Shaw CN (2013) Trabecular bone microstructure scales allometrically in the primate humerus and femur. Proc R Soc B 280: 20130172. 10.1098/rspb.2013.0172 PMC3619467 23486443

[46] Hunt KD (1991) Positional behaviour in the Hominoidea. Int J Primatol 12: 95–118.

[47] Carlson KJ, Patel BA (2006) Habitual use of the primate forelimb is reflected in the material properties of subchondral bone in the distal radius. J Anat 208: 659–670. 16761969 10.1111/j.1469-7580.2006.00555.x PMC2100237

[48] Marzke MW, Wullstein KL (1996) Chimpanzee and human grips: a new classification with a focus on evolutionary morphology. Int J Primatol 17: 117–139.

[49] Smith RJ, Jungers WL (1997) Body mass in comparative primatology. J Hum Evol 32: 523–559. 9210017 10.1006/jhev.1996.0122

[50] Tuttle RH, Watts DP (1985) The positional behavior and adaptive complexes of *Pan gorilla*. In: Kondo S, editor. Primate morphophysiology, locomotor analyses and human bipedalism. Tokyo: Tokyo University Press. pp. 261–288.

[51] Hunt KD (1992) Positional behaviour of *Pan troglodytes* in the Mahale Mountains and Gombe Stream National Parks, Tanzania. Am J Phys Anthropol 87: 83–105. 1736676 10.1002/ajpa.1330870108

[52] Doran DM (1996) Comparative positional behavior of the African apes. In: McGrew WC, Marchant LF, Nishida T, editors. Great ape societies. Cambridge: Cambridge University Press. pp. 213–224.

[53] Doran DM (1992) The ontogeny of chimpanzee and pygmy chimpanzee locomotor behavior: a case study of paedomorphism and its behavioural correlates. J Hum Evol 23: 139–157.

[54] Doran DM (1993) Comparative locomotor behavior of chimpanzees and bonobos: the influence of morphology on locomotion. Am J Phys Anthropol 91: 83–98. 8512056 10.1002/ajpa.1330910106

[55] Doran DM (1997) Ontogeny of locomotion in mountain gorillas and chimpanzees. J Hum Evol 32: 323–344. 9085185 10.1006/jhev.1996.0095

[56] Paoli G, Borgognini Tarli SM, Klír P, Strouhal E, Tofanelli S, et al (1993) Paleoserology of the Christian population at Sayala (Lower Nubia): an evaluation of the reliability of the results. Am J Phys Anthropol 92: 263–72. 8291618 10.1002/ajpa.1330920304

[57] Scherf H, Tilgner R (2009) A new high resolution-CT segmentation method for trabecular bone architectural analysis. Am J Phys Anthropol 140: 39–51. 19280676 10.1002/ajpa.21033

[58] Kothari M, Keaveny TM, Lin JC, Newitt DC, Genant HK, et al (1998) Impact of spatial resolution on the prediction of trabecular architecture parameters. Bone 22: 437–443. 9600776 10.1016/s8756-3282(98)00031-3

[59] Sode M, Burghardt AJ, Nissenson RA, Majumdar S (2008) Resolution dependence of the non-metric trabecular structure indices. Bone 42: 728–736. 18276202 10.1016/j.bone.2007.12.004 PMC2329672

[60] Isaksson H, Töyräs J, Hakulinen M, Aula AS, Tamminen I, et al (2010) Structural parameters of normal and osteoporotic human trabecular bone are affected differently by microCT image resolution. Osteoporos Int doi:10.1007/s00198-010-1219-0 10.1007/s00198-010-1219-0 20349043

[61] Treece GM, Prager RW, Gee AH (1999) Regularised marching tetrahedra: improved iso-surface extraction. Comput Graph 23: 583–598.

[62] Treece GM, Prager RW, Gee AH, Berman L (1999) Fast surface and volume estimation from non-parallel cross-sections for freehand 3-D ultrasound. Med Image Anal 3: 141–173. 10711996 10.1016/s1361-8415(99)80004-8

[63] Treece GM, Prager RW, Gee AH, Berman L (2000) Surface interpolation from sparse cross-sections using region correspondence. IEEE Trans Med Imaging 19: 1106–1114. 11204848 10.1109/42.896787

[64] Doube M, Kłosowski MM, Arganda-Carreras I, Cordeliéres F, Dougherty RP, et al (2010) BoneJ: free and extensible bone image analysis in ImageJ. Bone 47: 1076–1079. 20817052 10.1016/j.bone.2010.08.023 PMC3193171

[65] Pahr DH, Zysset PK (2009) From high-resolution CT data to finite element models: development of an integrated modular framework. Comput Methods Biomech Biomed Engin 12: 45–57. 18839383 10.1080/10255840903065399

[66] Pahr DH, Zysset PK (2009) A comparison of enhanced continuum FE with micro FE models of human vertebral bodies. J Biomech 42: 455–462. 19155014 10.1016/j.jbiomech.2008.11.028

[67] Whitehouse WJ (1974) The quantitative morphology of anisotropic trabecular bone. J Microsc 101: 153–168. 4610138 10.1111/j.1365-2818.1974.tb03878.x

[68] Raux P, Townsend PR, Miegel R, Rose RM, Radin EL (1975) Trabecular architecture of the human patella. J Biomech 8: 1–7. 1126969 10.1016/0021-9290(75)90037-8

[69] Odgaard A (1997) Three-dimensional methods for quantification of cancellous bone architecture. Bone 20: 315–328. 9108351 10.1016/s8756-3282(97)00007-0

[70] Zysset PK, Curnier A (1995) An alternative model for anisotropic elasticity based on fabric tensors. Mech Mater 21: 243–250.

[71] Zysset PK (2003) A review of morphology-elasticity relationships in human trabecular bone: theories and experiments. J Biomech 36: 1469–1485. 14499296 10.1016/s0021-9290(03)00128-3

[72] Pahr DH, Zysset PK (2008) Influence of boundary conditions on computed apparent elastic properties of cancellous bone. Biomech Model Mechanobiol 7: 463–476. 17972122 10.1007/s10237-007-0109-7

[73] Jungers WL, Falsetti AB, Wall CE (1995) Shape, relative size and size adjustments in morphometrics. Yrbk Phys Anthropol 38: 137–161.

[74] Coleman MN (2008) What does geometric mean, mean geometrically? Assessing the utility of geometric mean and other size variables in studies of skull allometry. Am J Phys Anthropol 135: 404–415. 18067122 10.1002/ajpa.20761

[75] Revell LJ (2012) Phytools: an R package for phylogenetic comparative biology (and other things). Methods Ecol Evol 3: 217–223.

[76] Revell LJ, Collar DC (2009) Phylogenetic analysis of the evolutionary correlation using likelihood. Evolution 63: 1090–1100. 19154380 10.1111/j.1558-5646.2009.00616.x

[77] Smaers JB, Dechmann DKN, Goswami A, Soligo C, Safi K (2012) Comparative analyses of evolutionary rates reveal different pathways to encephalization in bats, carnivorans, and primates. Proc Natl Acad Sci USA 109: 18006–18011. 23071335 10.1073/pnas.1212181109 PMC3497830

[78] Arnold C, Matthews LJ, Nunn CL (2010) The 10k trees website: A new online resource for primate phylogeny. Evol Anthropol 19: 114–118.

[79] Isler K, Kirk EC, Miller JMA, Albrecht GA, Gelvin BR, et al (2008) Endocranial volumes of primate species: scaling analyses using a comprehensive and reliable data set. J Hum Evol 55: 967–978. 18817943 10.1016/j.jhevol.2008.08.004

[80] Schilling A-M (2012) The trabecular structure of the primate wrist. M.Sc. Thesis, Università degli Studi di Pisa, Italy.

[81] Schilling A-M, Kivell TL (2013) The trabecular structure of the primate wrist. J Morph (in review). 10.1002/jmor.20238 24323904

[82] Tuttle RH (1969) Knuckle-walking and the problem of human origins. Science 166: 953–961. 5388380 10.1126/science.166.3908.953

[83] Loken B, Spehar S, Rayadin Y (2013) Terrestriality in the Bornean orang-utan (Pongo pygmeaus morio) and implications for their ecology and conservation. Am J Primatol (early view). 10.1002/ajp.22174 23784888

[84] Susman RL (1979) Comparative and functional morphology of hominoid fingers. Am J Phys Anthropol 50: 215–235. 443358 10.1002/ajpa.1330500211

[85] Inouye SE (1990) Variation in the presence and development of the dorsal ridge of the metacarpal head in African apes. Am J Phys Anthropol 81: 243.

[86] Inouye SE, Shea BT (2004) The implication of variation in knuckle-walking features for models of African hominoid locomotor evolution. J Anthropol Sci 82: 67–88.

[87] Badrian A, Badrian N (1977) Pygmy chimpanzees. Oryx 14: 463–472.

[88] Susman RL, Badrian NL, Badrian AJ (1980) Locomotor behavior of *Pan paniscus* in Zaire. Am J Phys Anthropol 53: 69–80.

[89] Stephens NB (2012) Trabecular bone architecture in the thumb of recent *Homo sapiens*, *Pan* and Late Pleistocene *Homo*. M.Sc. Thesis, University of London, UK.

[90] Lieberman DE (1996) How and why humans grow thin skulls: experimental evidence for systemic cortical robusticity. Am J Phys Anthropol 101: 217–236. 8893086 10.1002/(SICI)1096-8644(199610)101:2<217::AID-AJPA7>3.0.CO;2-Z

