# Supplementary material for: Trabecular Bone Structure Correlates with Hand Posture and Use in Hominoids
Source: PLoS One. 2013 Nov 14;8(11):e78781. doi: 10.1371/journal.pone.0078781 (PMC3828321; doi:10.1371/journal.pone.0078781)
Supplement: Table S1 — Complete study sample with raw data for each trabecular variable. (DOCX) [file pone.0078781.s001.docx]

**Supporting Information**

**Table S1. Study sample and mean absolute and trabecular thickness (Tb.Th), bone volume fraction (BV/TV) and degree of anisotropy (DA).**

| Specimen | Species | Side | Sex | Tb.Th | Scaled Tb.Th | BV/TV | DA |
| --- | --- | --- | --- | --- | --- | --- | --- |
| ZMB_83587 | *Gorilla gorilla* | L | F | 0.23 | 0.0124 | 0.21 | 0.32 |
| ZMB_83530 | *Gorilla gorilla* | R | M | 0.31 | 0.0134 | 0.23 | 0.36 |
| ZMB_83545 | *Gorilla gorilla* | R | M | 0.28 | 0.0145 | 0.29 | 0.21 |
| SMF_63976 | *Gorilla berengei* | L | U | 0.29 | 0.0144 | 0.26 | 0.31 |
| ZMB_38564 | *Hylobates agilis* | R | M | 0.15 | 0.0205 | 0.20 | 0.19 |
| ZMB_38566 | *Hylobates agilis* | R | M | 0.17 | 0.0260 | 0.16 | 0.24 |
| ZMB_38556 | *Hylobates agilis* | R | F | 0.12 | 0.0173 | 0.11 | 0.12 |
| ZMB_38562 | *Hylobates agilis* | R | U | 0.17 | 0.0275 | 0.15 | 0.04 |
| MRAC_15293 | *Pan paniscus* | L | F | 0.20 | 0.0170 | 0.28 | 0.27 |
| MRAC_15294 | *Pan paniscus* | L | M | 0.21 | 0.0179 | 0.28 | 0.26 |
| MRAC_20881 | *Pan paniscus* | L | M | 0.21 | 0.0170 | 0.28 | 0.28 |
| MRAC_27696 | *Pan paniscus* | R | M | 0.17 | 0.0124 | 0.23 | 0.29 |
| MRAC_27698 | *Pan paniscus* | L | F | 0.19 | 0.0149 | 0.26 | 0.24 |
| MRAC_29042 | *Pan paniscus* | R | F | 0.21 | 0.0158 | 0.31 | 0.22 |
| MRAC_29044 | *Pan paniscus* | R | M | 0.21 | 0.0150 | 0.31 | 0.30 |
| MRAC_29045 | *Pan paniscus* | L | F | 0.20 | 0.0148 | 0.27 | 0.24 |
| MRAC_29052 | *Pan paniscus* | R | M | 0.21 | 0.0153 | 0.32 | 0.27 |
| SMF_6779 | *Pongo abelii* | L | F | 0.21 | 0.0165 | 0.14 | 0.24 |
| SMF_6785 | *Pongo abelii* | L | M | 0.27 | 0.0189 | 0.24 | 0.02 |
| SMF_6999 | *Pongo abelii* | R | F | 0.15 | 0.0121 | 0.13 | 0.26 |
| SMF_24510 | *Pongo pygmaeus* | R | F | 0.27 | 0.0184 | 0.18 | 0.14 |
| SMF_74303 | *Pongo pygmaeus* | L | F | 0.27 | 0.0206 | 0.32 | 0.01 |
| SMF_84218 | *Pongo pygmaeus* | L | F | 0.20 | 0.0142 | 0.25 | 0.28 |
| ZMB_6947 | *Pongo pygmaeus* | L | M | 0.20 | 0.0163 | 0.18 | 0.12 |
| ZMB_6948 | *Pongo pygmaeus* | L | F | 0.19 | 0.0146 | 0.14 | 0.34 |
| ZMB_87092 | *Pongo pygmaeus* | R | U | 0.24 | 0.0161 | 0.20 | 0.24 |
| ZMB_38571 | *Symphalangus* | R | U | 0.18 | 0.0250 | 0.24 | 0.19 |
| ZMB_38587 | *Symphalangus* | L | U | 0.13 | 0.0166 | 0.18 | 0.19 |
| ZMB_38573 | *Symphalangus* | R | U | 0.21 | 0.0250 | 0.25 | 0.10 |
| ZMB_38583 | *Symphalangus* | R | F | 0.15 | 0.0207 | 0.14 | 0.15 |
| SMF_4104 | *Pan troglodytes* | L | F | 0.20 | 0.0133 | 0.28 | 0.18 |
| MPI_11903 | *Pan troglodytes* | L | M | 0.20 | 0.0124 | 0.26 | 0.27 |
| MPI_11789 | *Pan troglodytes* | L | M | 0.18 | 0.0124 | 0.23 | 0.24 |
| MPI_11781 | *Pan troglodytes* | L | M | 0.19 | 0.0128 | 0.20 | 0.28 |
| MPI_11778 | *Pan troglodytes* | L | F | 0.16 | 0.0112 | 0.24 | 0.21 |
| MPI _14996 | *Pan troglodytes* | L | F | 0.17 | 0.0123 | 0.21 | 0.30 |
| NHMW_K28_2 | *Homo sapiens* | R | F | 0.15 | 0.0134 | 0.15 | 0.25 |
| NHMW_J_2 | *Homo sapiens* | R | M | 0.17 | 0.0120 | 0.15 | 0.14 |
| NHMW_K_63 | *Homo sapiens* | L | M | 0.16 | 0.0112 | 0.16 | 0.20 |
| NHMW_K5_II | *Homo sapiens* | R | M | 0.16 | 0.0117 | 0.15 | 0.18 |
| NHMW_J_7 | *Homo sapiens* | R | F | 0.18 | 0.0158 | 0.12 | 0.08 |
| NHMW_K_41 | *Homo sapiens* | R | F | 0.18 | 0.0173 | 0.14 | 0.18 |
| NHMW_C1_31 | *Homo sapiens* | L | M | 0.13 | 0.0103 | 0.15 | 0.32 |
| NHMW_K24_2 | *Homo sapiens* | R | F | 0.17 | 0.0160 | 0.11 | 0.15 |
| NHMW_K18_2 | *Homo sapiens* | R | F | 0.16 | 0.0142 | 0.14 | 0.24 |
| NHMW_K_13_3 | *Homo sapiens* | R | M | 0.19 | 0.0163 | 0.18 | 0.32 |
