# Supplementary figures and images for: Trabecular Bone Structure Correlates with Hand Posture and Use in Hominoids
Source: PLoS One. 2013 Nov 14;8(11):e78781. doi: 10.1371/journal.pone.0078781 (PMC3828321; doi:10.1371/journal.pone.0078781)

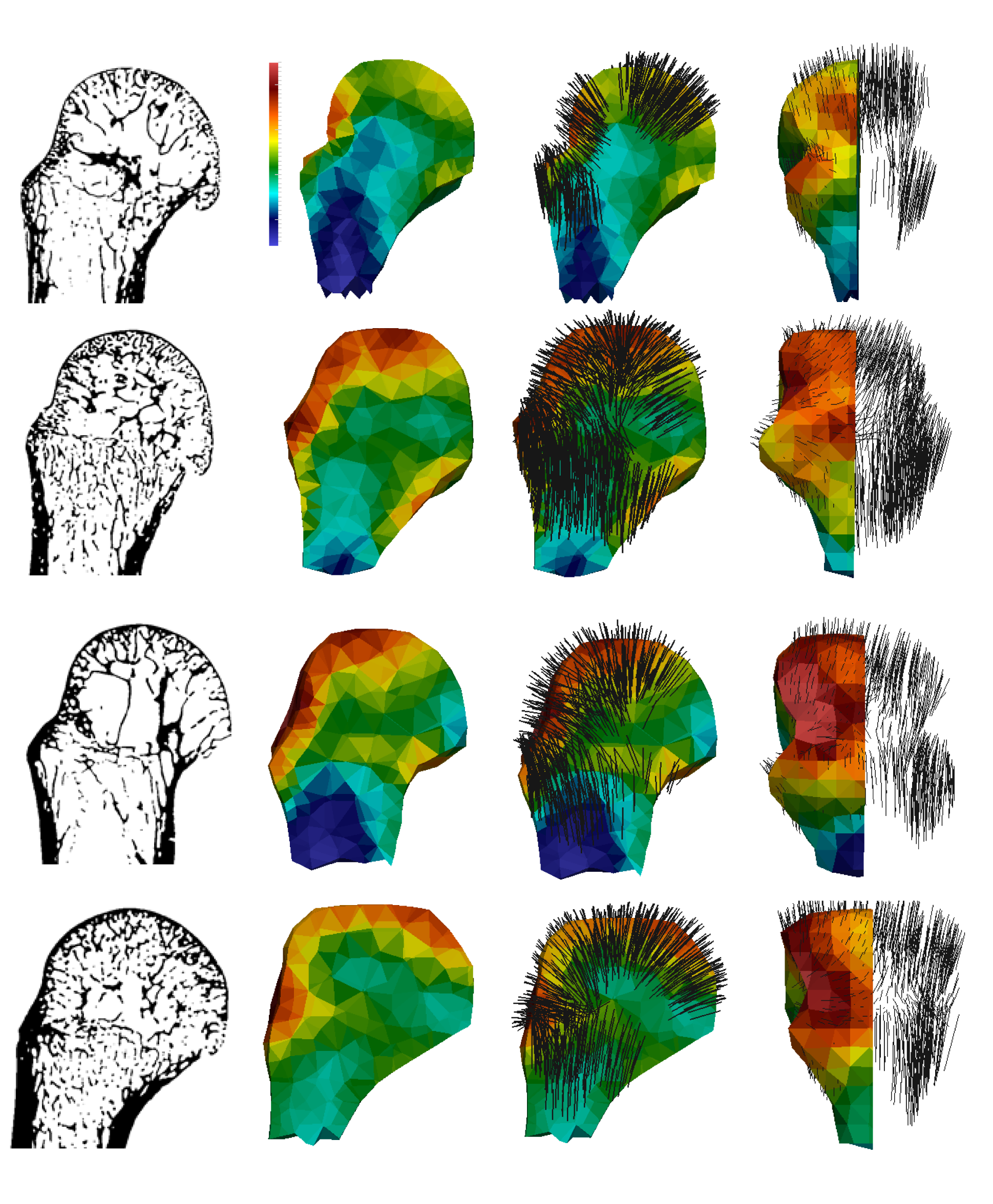

Supplement: Figure S1 — Gorilla sample, shown in a sagittal midline cross-sections of the third metacarpal head in four views. From left to right: cross section of the original scan; colour map of bone volume (BV/TV) distribution scaled to 0–0.45 (cortical bone has been removed); stiffness tensor maximum orientations superimposed on the bone volume colour map, the stiffness tensor is thresholded to E-modulus = 1000 Pa. Dorsal view of stiffness tensor maximum orientations superimposed on the bone volume colour map. (TIF) [file pone.0078781.s002.tif]

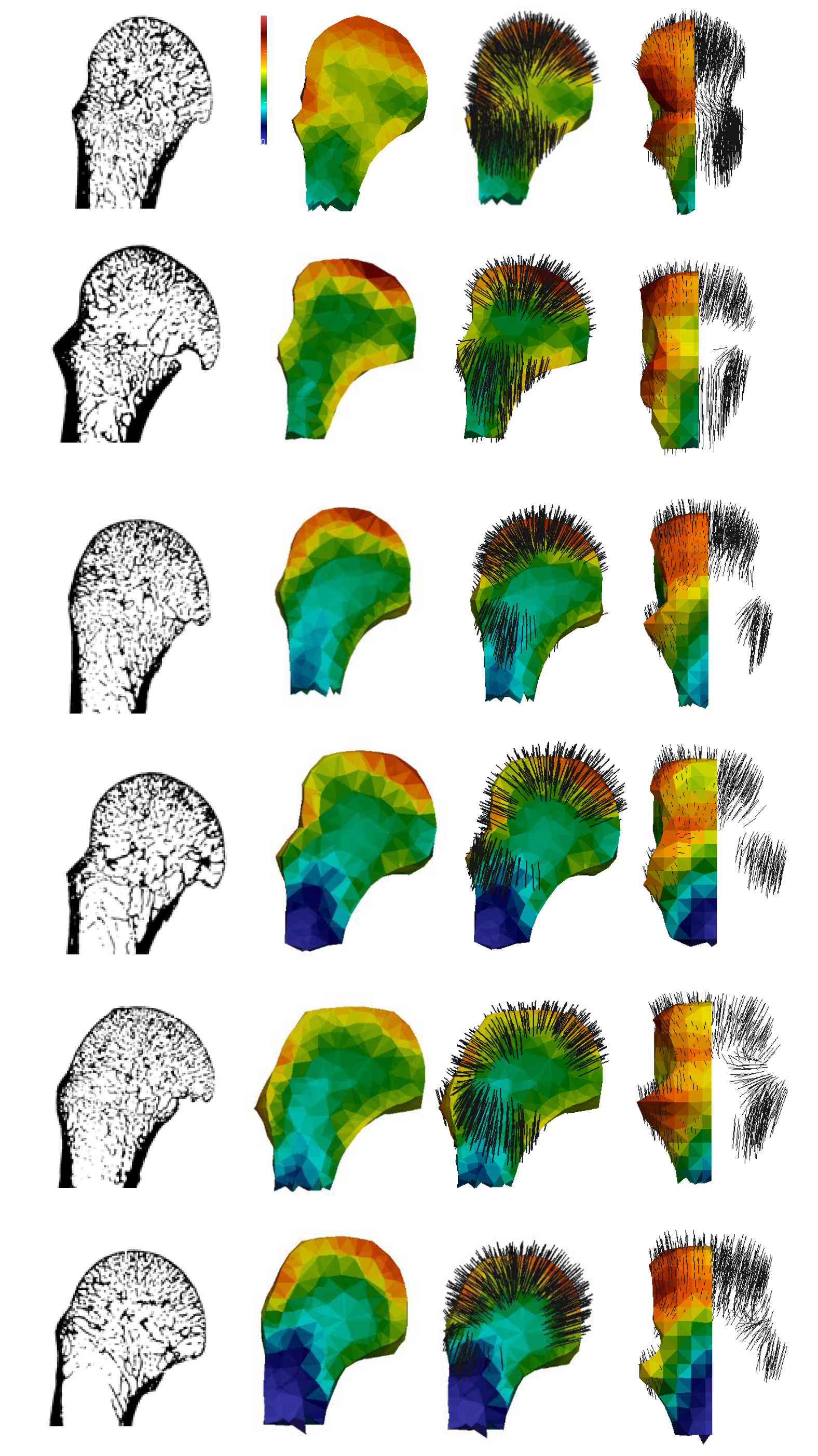

Supplement: Figure S2 — Pan troglodytes third metacarpal head sample, shown in same views as described in Figure S1. (TIF) [file pone.0078781.s003.tif]

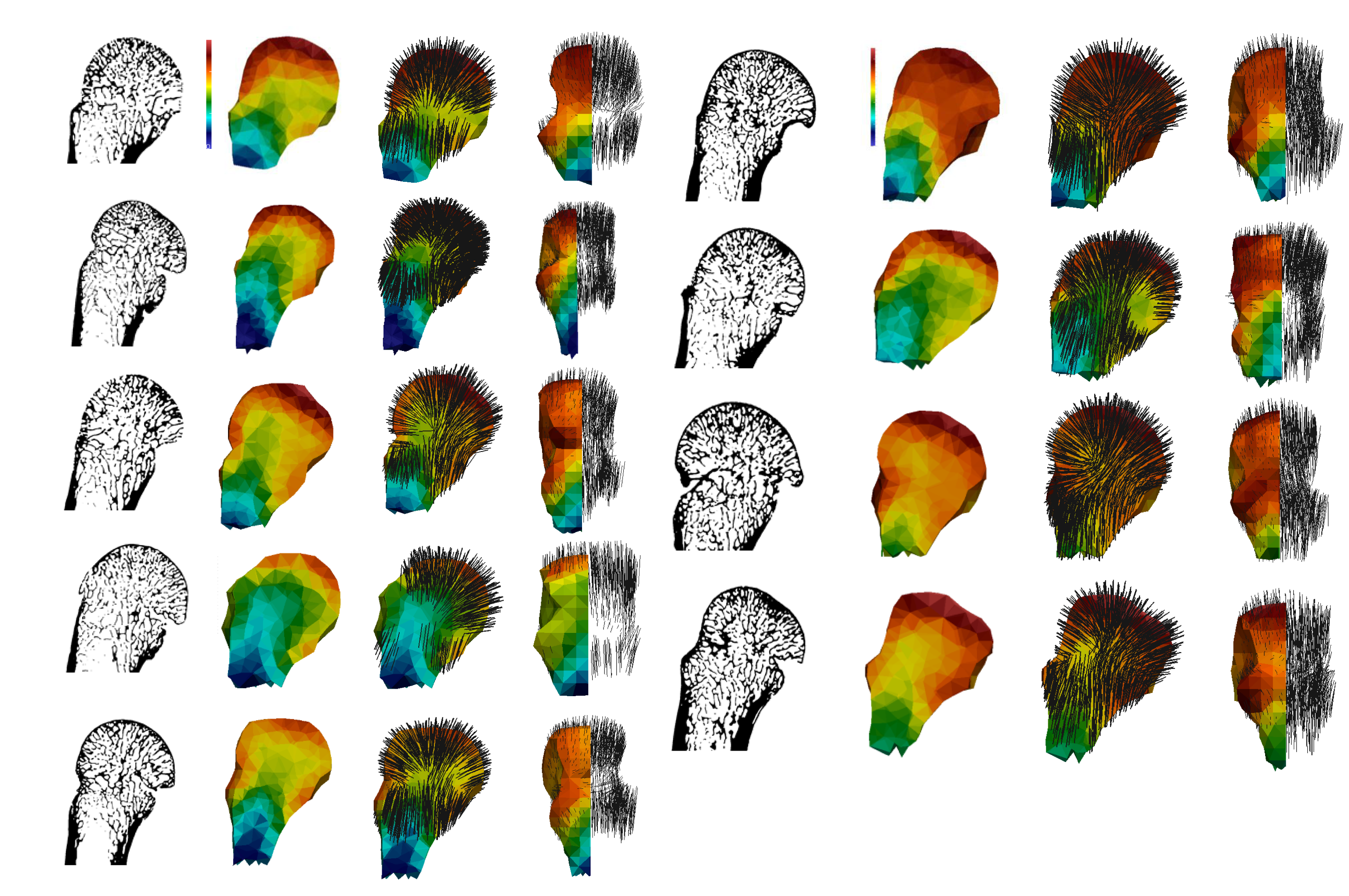

Supplement: Figure S3 — Pan paniscus third metacarpal head sample, shown in same views as described in Figure S1. (TIF) [file pone.0078781.s004.tif]

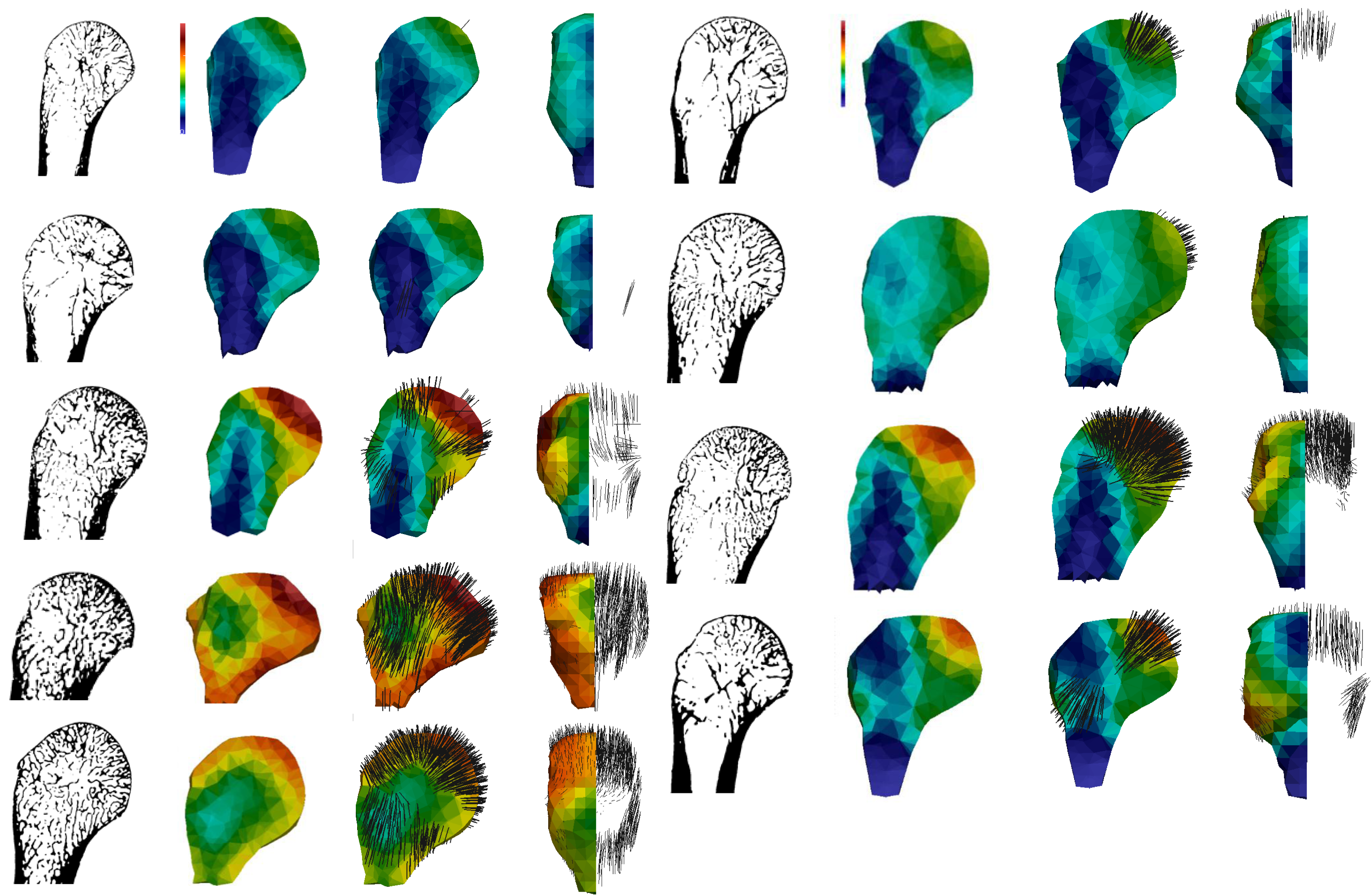

Supplement: Figure S4 — Pongo third metacarpal head sample, shown in same views as described in Figure S1. (TIF) [file pone.0078781.s005.tif]

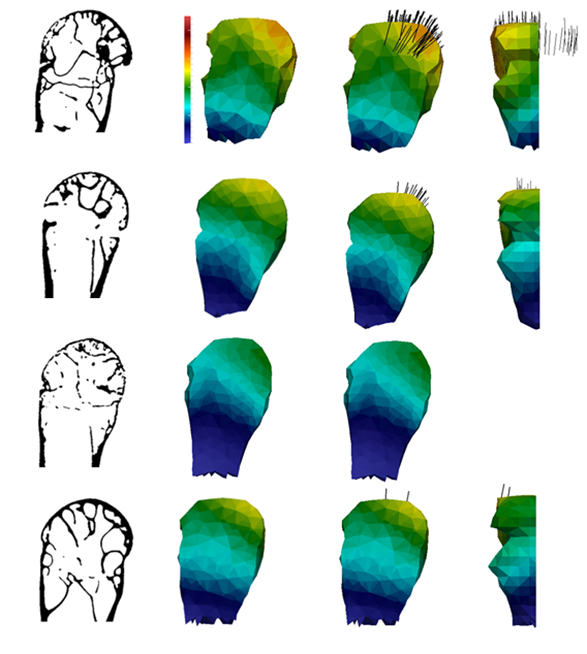

Supplement: Figure S5 — Hylobates agilis third metacarpal head sample, shown in same views as described in Figure S1. (TIF) [file pone.0078781.s006.tif]

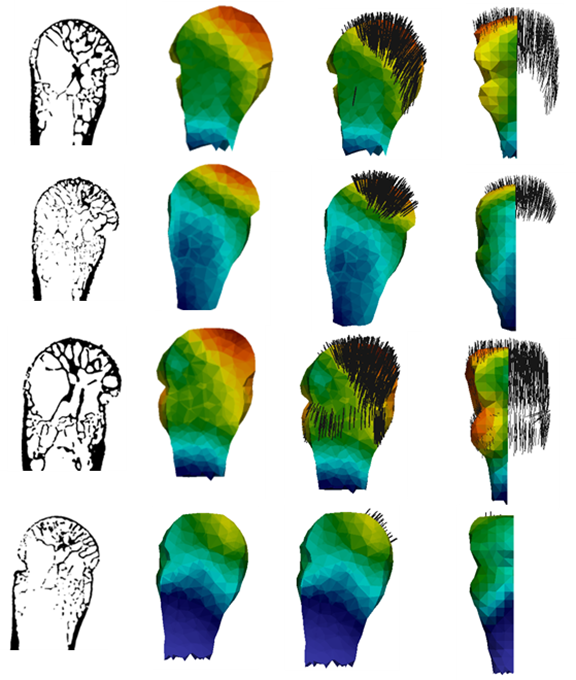

Supplement: Figure S6 — Symphalangus syndactylus third metacarpal head sample, shown in same views as described in Figure S1. (TIF) [file pone.0078781.s007.tif]

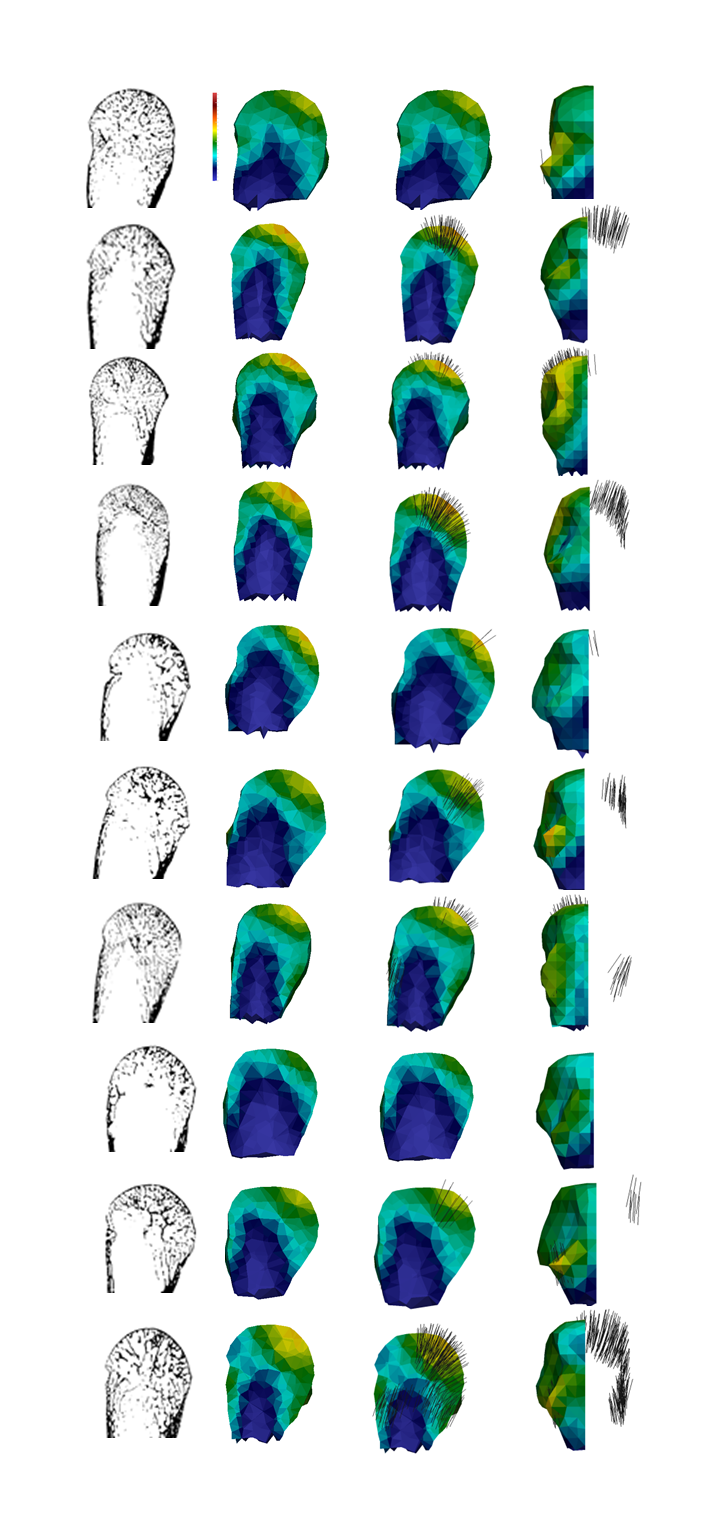

Supplement: Figure S7 — Homo sapiens third metacarpal head sample, shown in same views as described in Figure S1. (TIF) [file pone.0078781.s008.tif]
